# Evaluation of a quantitative PCR-based method for chimerism analysis of Japanese donor/recipient pairs

**DOI:** 10.1038/s41598-022-25878-9

**Published:** 2022-12-09

**Authors:** Keiji Minakawa, Satoshi Ono, Mao Watanabe, Yuka Sato, Saki Suzuki, Shou Odawara, Kinuyo Kawabata, Koki Ueda, Kenneth E. Nollet, Hideki Sano, Takayuki Ikezoe, Atsushi Kikuta, Kazuhiko Ikeda

**Affiliations:** 1 Department of Blood Transfusion and Transplantation Immunology, Fukushima Medical University Hospital, Fukushima, Japan; 2 Department of Blood Transfusion and Transplantation Immunology, Fukushima Medical University School of Medicine, 1 Hikarigaoka, Fukushima City, Fukushima 960-1295 Japan; 3 Department of Pediatric Oncology, Fukushima Medical University Hospital, Fukushima, Japan; 4 Department of Hematology, Fukushima Medical University School of Medicine, Fukushima, Japan

**Keywords:** Allotransplantation, Haematological diseases

## Abstract

Chimerism analysis is a surrogate indicator of graft rejection or relapse after allogeneic hematopoietic stem cell transplantation (HSCT). Although short tandem repeat PCR (STR-PCR) is the usual method, limited sensitivity and technical variability are matters of concern. Quantitative PCR-based methods to detect single nucleotide polymorphisms (SNP-qPCR) are more sensitive, but their informativity and quantitative accuracy are highly variable**.** For accurate and sensitive chimerism analysis, a set of KMR kits (GenDx, Utrecht, Netherlands), based on detection of insertions/deletions (indels) by qPCR, have been developed. Here, we investigated informativity and validated the accuracy of KMR kits in Japanese donor/recipient pairs and virtual samples of DNA mixtures representative of Japanese genetic diversity. We found that at least one recipient-specific marker among 39 KMR-kit markers was informative in all of 65 Japanese donor/recipient pairs. Moreover, the percentage of recipient chimerism estimated by KMRtrack correlated well with ratios of mixed DNA in virtual samples and with the percentage of chimerism in HSCT recipients estimated by STR-PCR/in-house SNP-qPCR. Moreover, KMRtrack showed better sensitivity with high specificity when compared to STR-PCR to detect recipient chimerism. Chimerism analysis with KMR kits can be a standardized, sensitive, and highly informative method to evaluate the graft status of HSCT recipients.

## Introduction

Allogeneic hematopoietic stem cell transplantation (allo-HSCT), including peripheral blood (PB) stem cell transplantation (PBSCT), bone marrow (BM) transplantation (BMT), and cord blood (CB) transplantation (CBT), can be a curative intervention for various hematologic diseases. However, allo-HSCTs often show comorbidity or mortality due to relapse of the hematologic disease, infections, graft-versus-host disease (GVHD), and graft failure associated with graft rejection or poor graft function^1^. Sufficient hematopoietic and immune reconstitution by replacement of recipient-derived cells with donor cells is critical for successful allo-HSCT^2,3^.

Chimerism analysis assesses proportions of hematopoietic cells from the donor (donor chimerism) and recipient (recipient chimerism), and it is used as a confirmation of engraftment and as a surrogate indicator for graft rejection or relapse^4,5^. The major techniques of chimerism analysis are PCR for short tandem repeat (STR) profiles of DNA (STR-PCR), and detection of single nucleotide polymorphisms (SNPs) with real-time quantitative PCR (qPCR)-based methods (SNP-qPCR)^4–7^. Although STR-PCR is the predominant method, technical variability among laboratories is pronounced, and the detection limit for recipient chimerism is not very low, ranging from 1 to 10% among studies^7–13^. SNP-qPCR is a fast and sensitive method to detect recipient chimerism below 1%, but there are issues such as limited informativity, inconsistent quantitative accuracy, false positive results, and technical variations^4,12^. Thus, chimerism analysis lacks methodological standardization and might not discriminate between donor and recipient cells with sufficient reliability^4–7,12,13^.

Recently, KMR kits (GenDx, Utrecht, Netherlands) have been developed as a set of standardized products prepared for chimerism analysis using a real-time qPCR-based assay. Appropriate markers can be screened from among ready-to-use primers for 39 different chromosomal locations in the KMRtype Core and Extended kits, followed by post-allo-HSCT chimerism monitoring with KMRtrack using the primer(s) selected by the KMRtype kits. The 39 KMR markers, which are commercially available as CE-IVD markers, detect specific insertions/deletions (indels) that consist of multiple nucleotides instead of single nucleotides as in SNP-qPCR. As for methodologies and standardization in chimerism analysis, genetic diversity and distribution of indels may differ among ethnic groups of donors and recipients, for which reason KMR kits should be validated in specific contexts. Here, we assessed the informativity and accuracy of chimerism monitoring using KMR kits on samples drawn from an ethnic Japanese cohort.

## Materials and methods

### Samples and study design

Genomic DNA was extracted using the QuickGene DNA whole blood kit S (KURABO, Osaka, Japan) or the SMITEST EX-R&D (MBL, Tokyo, Japan) from 130 individuals comprising 65 Japanese donor/recipient pairs who underwent allo-HSCT at Fukushima Medical University Hospital between January 2009 and March 2021 (Table 1). Most of the PBSCT grafts were from related donors, whereas BM grafts mainly derived from unrelated donors. Each CB graft was sourced from a single unrelated donor, following Japan Cord Blood Bank Network/Japanese Marrow Donor Program standards, as almost all CBTs in Japan have used grafts from single donors^14,15^.

**Table 1:** Donor/recipient pairs.

Variables	*N*
**Donor sources (related/unrelated)**	
BM	2/17
PB	31/1
CB	0/14
Total	33/32
Sex (matched/mismatched)	39/26
**HLA mismatch**	
Related BMT/PBSCT	33
0/8	3
1/8	3
≥ 2/8 (Haploidentical)	27
Unrelated BMT/PBSCT	18
0/8	10
≥ 1/8	8
Unrelated CBT	14
0/8	0
≥ 1/8	14

To screen for informative markers that can distinguish donor and recipient cells, pre-allo-HSCT DNA samples purified from pairs of donors (PB, BM, or CB cells) and recipients (PB cells or buccal swab) were used in KMRtype Core and, as necessary, KMRtype Extended kits (GenDx). Virtual samples of DNA mixtures with known concentrations obtained from healthy Japanese volunteers were used to validate KMRtrack (GenDx), a tool intended for chimerism monitoring. Concentration of DNA was measured by the absorbance at 260 nm in a spectrophotometer (NanoDrop One^C^, Thermo Fisher Scientific, MA, USA). DNA samples with > 1.8 260/280 nm ratio were mixed carefully for generation of virtual samples. We also tested post-allo-HSCT DNA samples from recipient PB or BM cells. In these samples, values of recipient chimerism obtained by KMRtrack (KMR recipient chimerism) were compared with those of STR-PCR or in-house SNP-qPCR, which had been performed as routine examinations for clinical practice.

Whenever possible, written informed consent was obtained from enrollees, and the opt-out method was applied in some cases for retrospective study of anonymized data. This study was approved by the Ethics Committee of Fukushima Medical University (Approval number 2020-030), which is guided by local policy, national law, and the Declaration of Helsinki.

### STR-PCR and in-house SNP-qPCR

For STR-PCR, 20 STR markers (D18S1270, D12S391, D20S161, D11S488, D14S608, D10S2325, D8S306, D9S304, D8S1179, D8S639, D19S253, D16S3253, D21S1437, FGA, D5S818, SE33, TH01, VWF, Penta E, D18S51) were amplified using a GeneAmp PCR System 9700 (Thermo Fisher Scientific, Waltham, MA, USA) as described previously^3,16,17^. In-house SNP-qPCR was performed with the sets of primers and probes specific for 5 SNPs (rs2385512, rs3769393, rs748235, rs1386718, rs12438539)^18^, using a QuantStudio 3, followed by analysis with QuantStudio Design & Analysis Software v1.4.3 (Thermo Fisher Scientific).

### KMR kits

Analysis with KMR kits, which are ready-to-use products prepared for real-time qPCR-based assay, was performed using 10 ng aliquots of DNA from each sample according to manufacturer’s instructions. These kits consist of KMRtype and KMRtrack for pre-allo-HSCT screening and chimerism monitoring after allo-HSCT, respectively. KMRtype Core contains 10 multiplexed primer mixes for 30 markers to distinguish donor-derived and recipient-derived cells for pre-allo-HSCT screening; KMRtype Extended contains 9 additional markers for cases without an appropriate marker in the KMRtype Core set (Supplemental Table 1). KMRtrack monitors post-allo-HSCT chimerism using a marker determined by pre-allo-HSCT screening with one or both KMRtype kits. KMRtype and KMRtrack assays contain a Reference assay that is used as internal reference for relative quantification (REF901). The Reference assay is an oligonucleotide, with a fluorophore, that specifically targets an invariant control or housekeeping gene present in DNA samples of all humans. The relative presence of the informative markers is determined in both the pre- and post-transplant samples by comparing the Cq value of the marker to the Cq value of the Reference assay (∆Cq). The change between the two time points, pre- and post-transplant, is then calculated (∆∆Cq). This is subsequently converted to a ratio or percentage. QuantStudio 6 Flex (Thermo Fisher Scientific) was used for qPCR. Results obtained by the KMRtype or KMRtrack kits were analyzed with KMRengine software (GenDx), which determines positivity or negativity and proportions of donor- and recipient-derived cells, according to the ∆∆Cq values. In cases of Cq values ranging outside of predicted levels, KMRengine software judges the result as an “atypical amplification” to exclude non-specific amplification (Supplemental Fig. 1).

### Statistical analysis

The unpaired *t* test or Kruskal–Wallis test was used for comparisons of 2 or 3 groups, respectively. The relationship of proportions of recipient cells between the previous results of STR-PCR or in-house SNP-qPCR and those of the KMRtrack kit was analyzed with correlation coefficients and linear regression. Bland–Altman analysis was also used to compare results of the KMRtrack kit with previous methods.

## Results

### KMRtype kits for Japanese allo-HSCT donors/recipients

**Figure 1:**
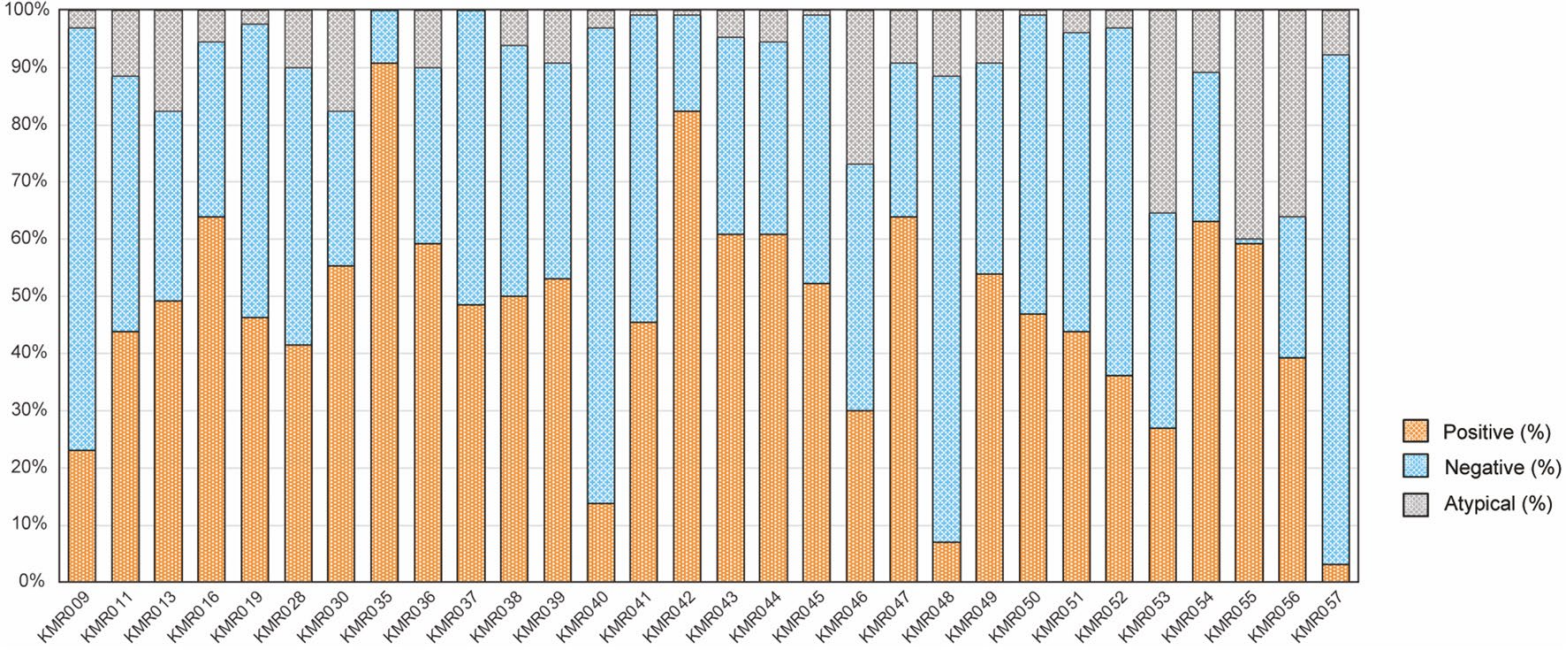
Positivities/negativities of markers in KMRtype Core kit. Proportions of individuals positive or negative for each marker by typing with the KMRtype Core kit. “Atypical” indicates PCR reaction with either a lower or higher Cq than the preset values.

**Figure 2:**
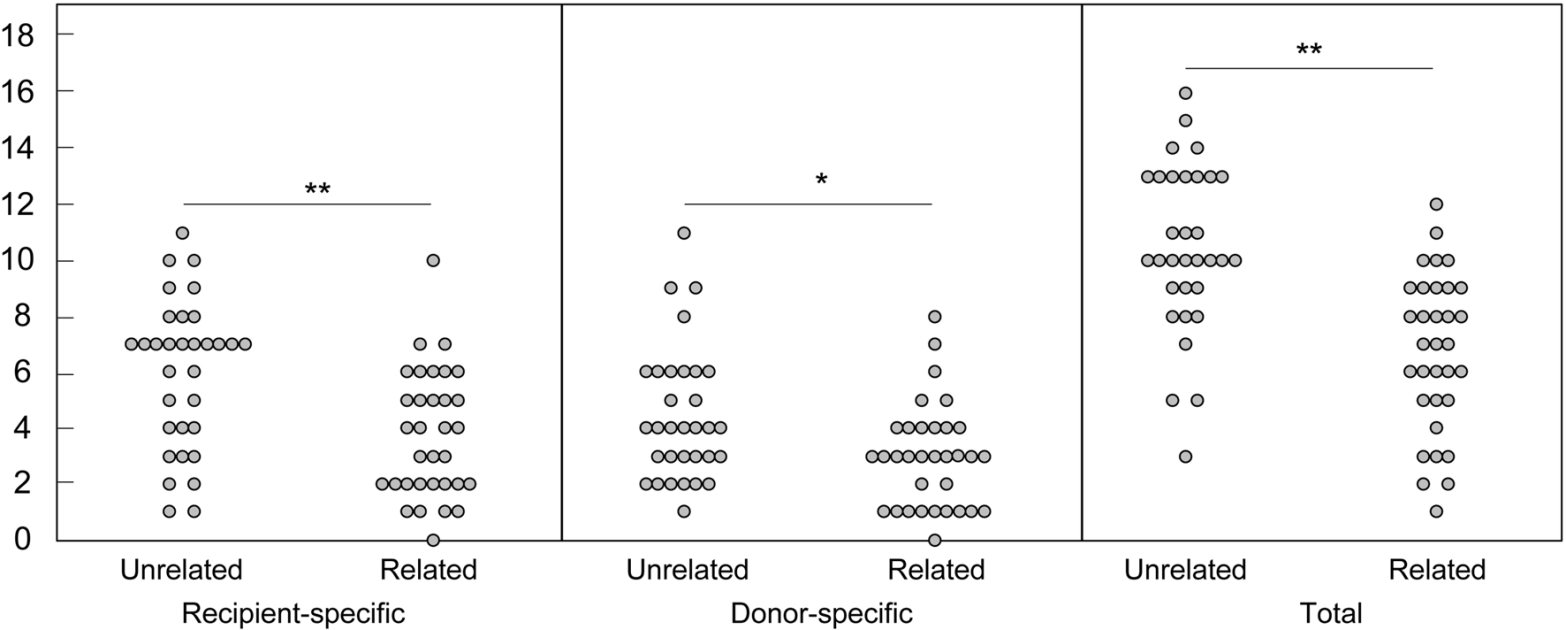
Numbers of recipient-specific, donor-specific, and total informative markers in KMRtype Core kit for each donor/recipient pair. *: *p* < 0.05, **: *p* < 0.01.

We investigated whether the 30 markers in KMRtype Core can distinguish between donor and recipient cells in samples from 65 Japanese donor/recipient pairs of allo-HSCT (Table 1), consisting of 130 individuals. Among 30 markers in KMRtype Core, each marker showed either positivity or negativity for at least one individual, and 24 markers were informative for over 20% of donor/recipient pairs (Fig. 1, Supplemental Fig. 1, Supplemental Table 1). Accordingly, we could distinguish all the pairs using at least one marker, indicating high informativity from KMRtype Core marker set for chimerism analysis of Japanese donor/recipient pairs (Fig. 2). Next, we investigated if KMRtype Extended could add informativity to the 23 donor/recipient pairs with no more than 3 recipient-specific markers in KMRtype Core (Supplemental Fig. 2, Supplemental Table 2), because use of multiple recipient-specific markers is preferable for detection of recipient cells in qPCR-based methods^5,7,12,19^. A recipient-specific marker in the KMRtype Extended kit was informative in the one recipient (UID4533) without any recipient-specific marker in the KMRtype Core kit, resulting in 100% informativity of the KMR kits with recipient-specific markers for the 65 Japanese donor/recipient pairs. However, even with both KMRtype Core and Extend kits, 6 pairs had only one recipient-specific marker (Supplemental Table 2). Thus, 59 per 65 (90.8%) pairs were informative with ≥ 2 recipient-specific markers. There were some markers that frequently showed atypical results with indeterminate PCR amplification.

Regarding donor-recipient HLA matching in allo-HSCTs, 27 out of 33 related pairs were haploidentical with ≥ 2/8 mismatches, while numbers of HLA mismatches were ≤ 1/8 in most of the unrelated pairs (Table 1). Despite fewer differences in the HLA alleles of unrelated pairs, the numbers of recipient-specific, donor-specific, and total informative markers were significantly greater in unrelated pairs compared with those of related pairs (Fig. 2).

### Accuracy and reproducibility of KMRtype kit for detection of recipient cells

**Figure 3:**
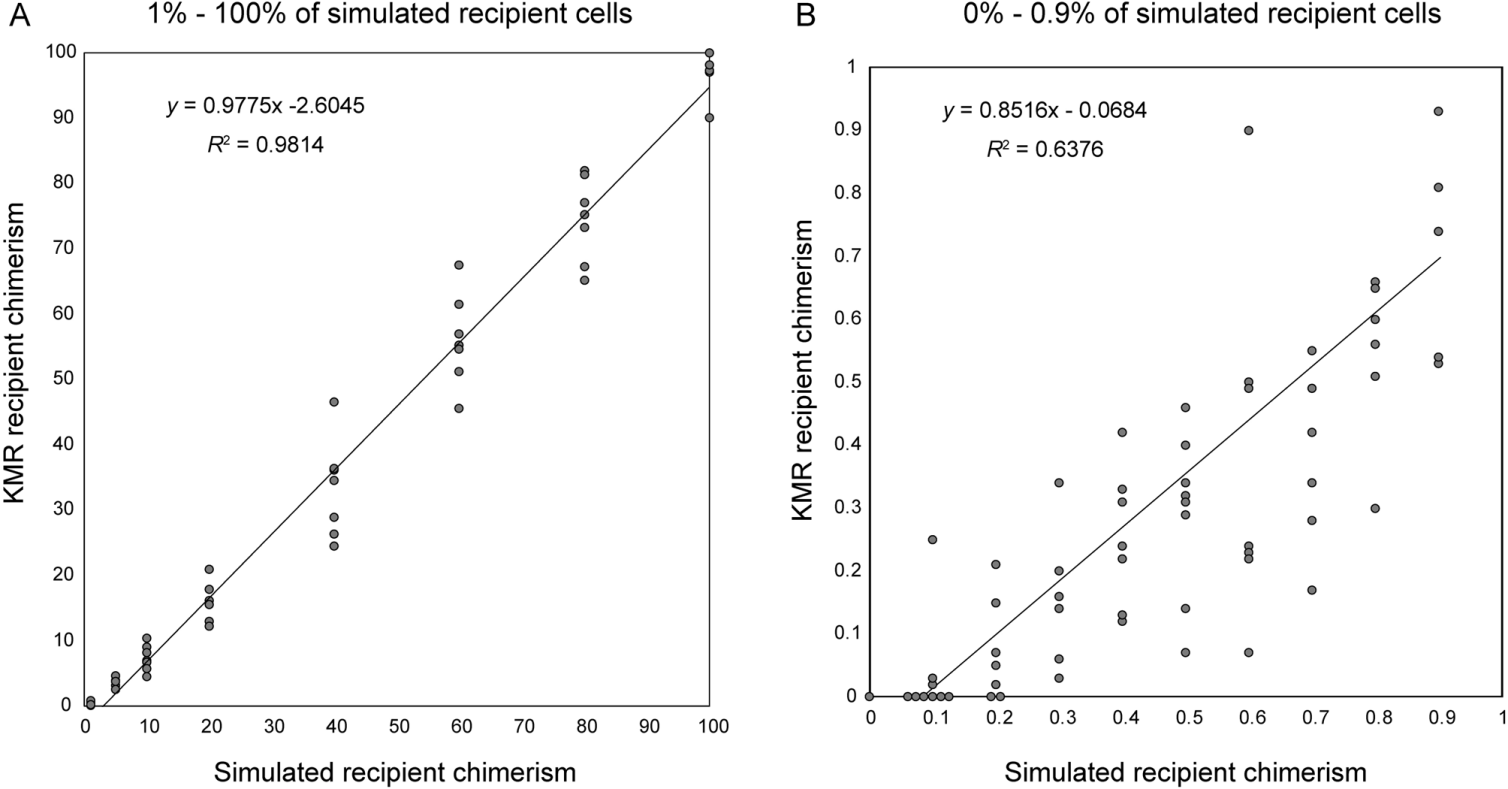
Correlation of KMR and simulated chimerism in virtual samples. Chimerism was evaluated by KMRtrack in virtual samples containing mixtures of DNA from 2 individuals. DNA mixtures with (**A**) 1%, 5%, 10%, 20%, 40%, 60%, 80%, and 100%, or (**B**) 0%, 0.1%, 0.2%, 0.3%, 0.4%, 0.5%, 0.6%, 0.7%, 0.8%, and 0.9%, of simulated recipient cells were prepared according to DNA concentrations. The recipient chimerisms of these DNA mixtures were evaluated by KMRtrack using the 7 markers with highest informativity to distinguish Japanese donor/recipient pairs.

Seeking a group of markers that can cover over 95% of donor/recipient pairs in the KMRtype Core kit, we found that 7 with the highest informativity (KMR019, KMR028, KMR037, KMR041, KMR045, KMR049, KMR051) could distinguish 62 of 65 pairs (95.4%). With these 7, we tested the accuracy and reproducibility of KMR markers for estimating ratios of recipient cells with the KMRtrack kit, using virtual samples with known concentrations of mixed DNA from 2 individuals. For samples with ≥ 1% of virtual recipient DNA, values of KMR chimerism determined by these markers correlated highly with ratios of DNA concentrations from 2 individuals, mixed to simulate recipient chimerism (*r* = 0.991, *p* < 0.001, Fig. 3A). Regarding the capability of detecting a low proportion of recipient cells, we evaluated samples with < 1% of 10 ng virtual recipient DNA. KMRtrack detected the minor recipient chimerism in these samples, with a lower correlation coefficient between KMR values and the actual degree of simulated chimerism (*r* = 0.798, *p* < 0.001, Fig. 3B) than in the samples with ≥ 1% of virtual recipient DNA. The samples with ≥ 1% of virtual recipient DNA also showed smaller coefficient of variation (CV) values of aliquots than those with < 1% of virtual recipient DNA (Supplemental Table 8). Over 2 CV values were noted in 2 per 56 (3.5%) samples with ≥ 1% of virtual recipient DNA, while 24 per 67 (35.8%) samples with < 1% of virtual recipient DNA showed over 2 CV values. Recipient chimerism was detected in all the samples with ≥ 0.3% of virtual recipient DNA, whereas amplification was not observed in half (8 of 16) samples with ≤ 0.2% of virtual recipient DNA (Fig. 3A-B). Thus, accuracy and reproducibility of the KMR track kit were better to detect ≥ 1% than < 1% recipient DNA, although this kit can certainly detect 0.1–1% recipient DNA in most of samples. KMR estimates of recipient chimerism tended to be lower than the simulated degree of recipient chimerism.

### Comparisons of KMRtrack with other methods of chimerism monitoring

**Figure 4:**
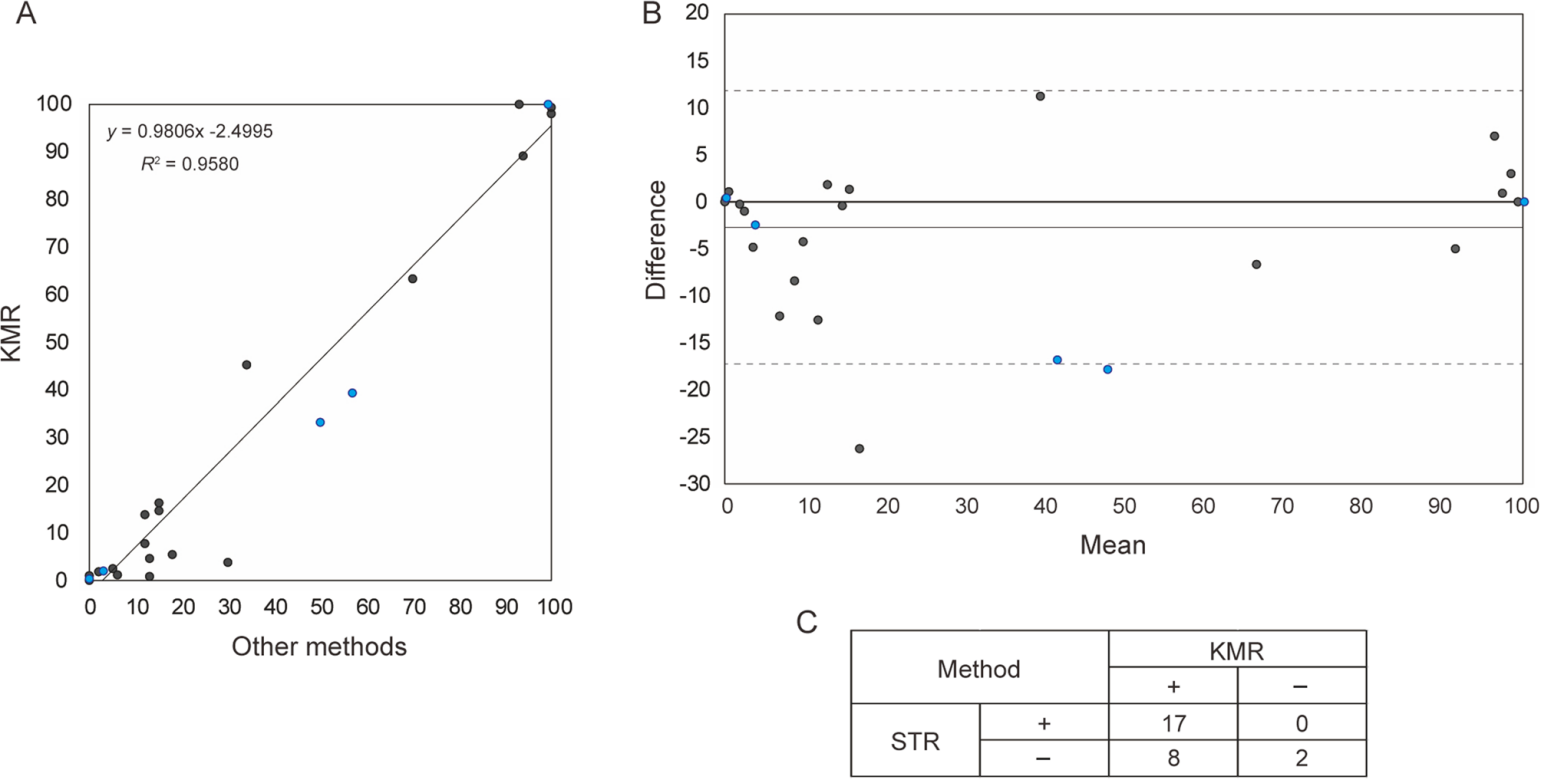
Recipient chimerism determined by KMR and conventional methods. (**A**) Correlation between recipient chimerism evaluated with KMRtrack and those with STR-PCR or in-house SNP-qPCR. Values of recipient chimerism are shown as percentages in each sample. (**B**) Bland–Altman analysis to compare percentage estimates of chimerism by KMR versus STR-PCR/in-house SNP-qPCR. Dotted lines indicate mean ± 2 SD (standard deviation). (**C**) Positivity/negativity of recipient chimerism determined by KMRtrack (KMR) and STR-PCR (STR). (**A, B**) Black dots indicate the values measured by KMRtrack and STR-PCR, while blue dots were values from KMRtrack and in-house SNP-qPCR.

We studied the quality of KMRtrack with recipient-specific markers in a clinical setting with the aforementioned most informative 7 markers. We randomly chose 32 post-allo-HSCT DNA samples, which were evaluable using these 7 markers, including 11 samples with no recipient chimerism (complete donor chimerism) and 21 with mixed chimerism according to STR-PCR or in-house SNP-qPCR with recipient-specific markers previously tested in our laboratory. The values of KMR recipient chimerism correlated well with those of STR-PCR/in-house SNP-qPCR recipient chimerism (*r* = 0.978, *p* < 0.001, Fig. 4A). Bland–Altman analysis showed similarity in chimerism estimates between KMR and STR-PCR/in-house SNP-qPCR, with a minor bias of slightly low levels of recipient chimerism according to KMR (Fig. 4B). We next explored differences in the positivity of identical samples for recipient chimerism between STR-PCR and KMRtrack. KMRtrack detected minor recipient chimerism (0.19–1.05%) in 8 of 10 samples for which no recipient chimerism was detected by STR-PCR, whereas STR-PCR did not show positivity in any sample that was negative for KMR recipient chimerism (Fig. 4C, Supplemental Fig. 3), indicating higher sensitivity with KMRtrack.

**Figure 5:**
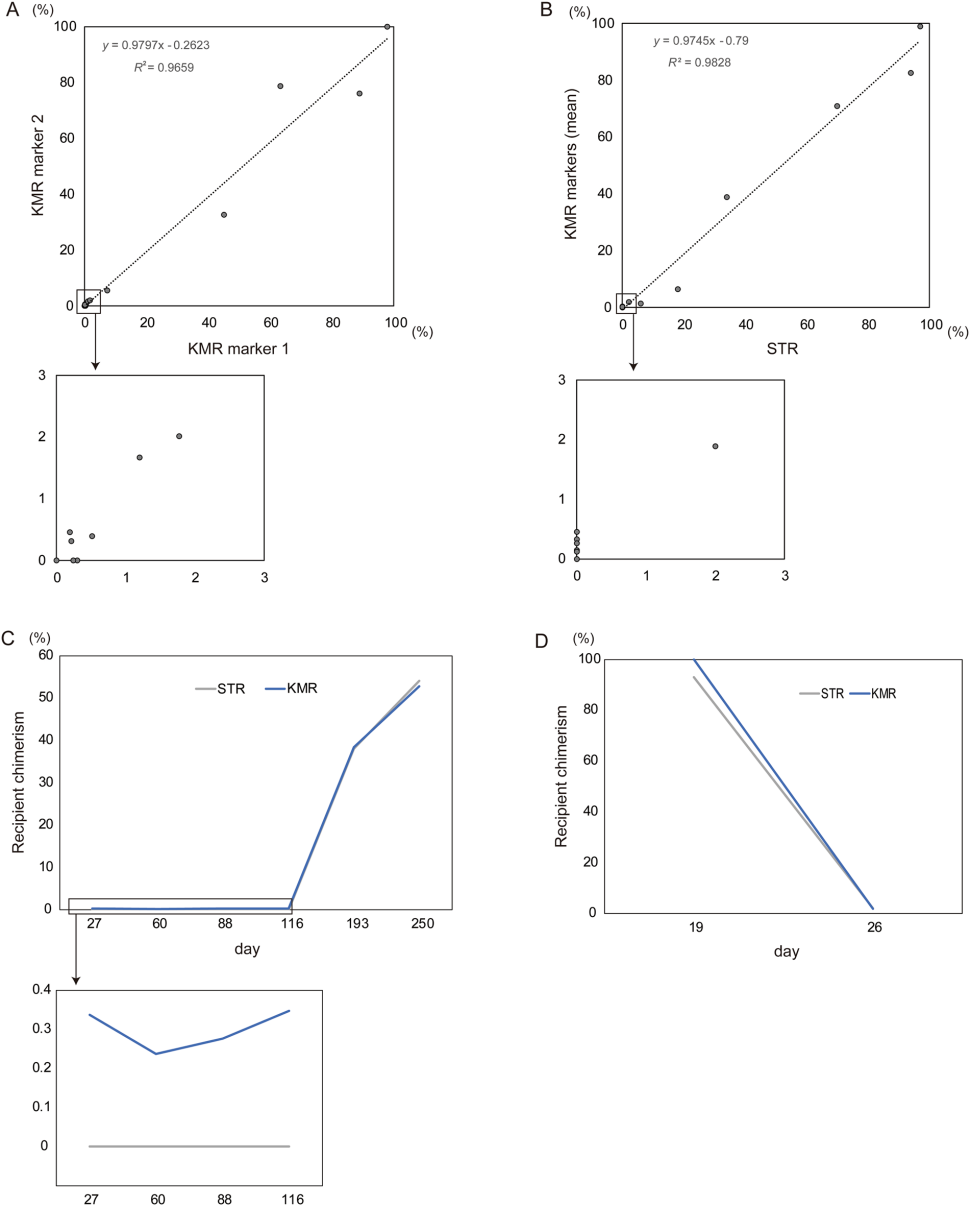
Validation of chimerism monitoring by KMR method. (**A**) Correlation between recipient chimerism evaluated with one KMR marker and another. (**B**) Correlation between means of recipient chimerism levels in couples of KMR markers and results of STR-PCR method. (**A, B**) Levels of recipient chimerism are shown as percentages in each sample. (**C, D**) Kinetics of changing chimerism in patients with relapsing (**C**) or engrafting (**D**). KMR results are indicated as means of multiple markers, except day 19 in which sample was not available for additional KMR examination due to the low cell number. Detailed data are depicted in Supplemental Tables 3, 4, 5.

When we used 2 different KMR markers, recipient chimerism levels well correlated with each other regardless of the degree of recipient chimerism levels (Fig. 5A, Supplemental Table 3). Mean chimerism levels of 2 KMR markers were well correlated with those of STR-PCR, although low levels of recipient chimerism was detected only in KMR (Fig. 5B). Multiple KMR markers could also demonstrate increasing or decreasing chimerism, which reasonably corresponded to disease status and results of STR-PCR, during clinical course (Fig. 5C-D, Supplemental Tables 4, 5). We then set KMRtrack to evaluate 8 samples that had been tested by in-house SNP-qPCR with donor-specific markers, because recipient-specific markers were absent. Three samples showed similarly low values of recipient chimerism between in-house SNP-qPCR with a donor-specific marker (0% each) and KMRtrack (0–0.46%). In the other 5 samples, including 2 couples of samples from 2 patients obtained at different time points (Supplemental Table 3), multiple KMR markers showed low values of recipient chimerism (0–0.44%), although in-house SNP-qPCR with donor-specific markers indicated mixed chimerism (values of recipient chimerism: 18–30%). Considering that multiple KMR markers showed similarly low recipient chimerism levels and all of these samples were derived from allo-HSCT recipients in persistent complete remission of hematologic cancers, it is likely that results of KMRtrack were more accurate than those of in-house SNP-qPCR with donor-specific markers.

## Discussion

For chimerism analysis, ≥ 12 markers are recommended to attain > 95% informativity in STR-PCR^13^. In SNP-qPCR, informativity is more limited than in STR-PCR and does not reach 100% in most cases, although it depends on the number and distribution of markers^13,20^. By contrast, we found at least one recipient-specific marker in the 39 markers of the KMR kits for all of 65 Japanese donor/recipient pairs, indicating 100% informativity in this population. Availability of a recipient-specific marker from a large set of 39 KMR-kit markers is important, because the use of a recipient-specific marker is recommended for chimerism monitoring with SNP-qPCR^5,12,19^. In fact, we found more accurate results with KMRtrack than with in-house SNP-qPCR using a donor-specific marker in cases without recipient-specific markers by this method. Moreover, the choice of marker for STR-PCR is often complicated because of stutter interference and preferential amplification in the PCR reaction, and commercially available kits for STR-PCR are generally designed for forensic identification and not optimized for chimerism analysis^7^. Regarding SNP-qPCR methods, it has been reported that using < 10 markers results in 80%-97% informativity, suggesting the need for a larger set of markers^13,20^. Additionally, fluorescence in situ hybridization with sex chromosomes is usable only for sex-mismatched donor/recipient pairs^4^. Therefore, the high informativity of premixed markers may be an advantage of the KMR kits.

The numbers of recipient-specific, donor-specific, and overall total of informative markers in the KMRtype Core kit were significantly greater for unrelated pairs than for related pairs, probably reflecting smaller disparities within families than among unrelated members of a general population. This was even the case for unrelated pairs with matched HLA compared to related HLA-haploidentical pairs with greater disparities in their HLA alleles. High genetic similarity outside the HLA region in related HLA-haploidentical pairs might contribute to successful HLA-haploidentical allo-HSCT^21–24^. Likewise, a recent study by Tyler et al. from the United States also showed that there were more informative markers for unrelated pairs than related pairs in the 30 KMR markers^25^, although HLA compatibility was not described. In their study, 8 of 60 donor/recipient pairs (13.3%) had ≤ 2 recipient-specific markers, whereas in our cohort, 6 of 65 donor/recipient pairs (9.2%) had ≤ 2 recipient-specific markers from the KMRtype Core kit. Our results suggest that KMR kit markers enable us to perform appropriate chimerism analysis in a wide variety of donor/recipient pairs. A limitation is that although use of multiple recipient-specific markers is recommended for chimerism monitoring, one unrelated and 5 related pairs had only one recipient-specific marker by using both KMRtype Core and Extended kits. This may be consistent with a recent study that showed at least 40 markers are required to distinguish a large number of the pairs^26^. There is another issue that some markers frequently showed atypical results which could not determine chimerism.

Next, assessment of the KMRtrack kit using virtual pairs consisting of DNA from 2 different individuals showed good correlation between measured and simulated degrees of chimerism, with sensitivity high enough to detect thresholds of 0.1%-0.3% of an amount as small as 10 ng of DNA. Likewise, values of KMR recipient chimerism correlated well with values from previous chimerism analyses performed with STR-PCR or in-house SNP-qPCR. Although KMR recipient chimerism values tended to be lower than the simulated recipient chimerism values of virtual samples or STR-PCR/in-house SNP-qPCR chimerism in post-allo-HSCT samples, KMRtrack detected minor recipient chimerism even in post-allo-HSCT samples for which STR-PCR did not detect recipient chimerism. This indicates better sensitivity of KMRtrack versus STR-PCR, together with the assessment of virtual samples, described above. In contrast, STR-PCR did not detect recipient chimerism in the post-allo-HSCT samples that were negative for KMR recipient chimerism. These findings suggest sufficient quality, sensitivity, and specificity of the KMRtrack kit for chimerism monitoring. However, a limitation is that positivity of the KMRtrack kit does not distinguish between homozygous and heterozygous state of the marker. In addition, there was a certain variability among KMR markers in chimerism monitoring (Fig. 3), indicating that using multiple markers and reporting their mean increase accuracy of chimerism results, as it has been recommended^7^. For donor/recipient pairs with only one marker, we should be aware that accuracy is an important but challenging characteristic.

The requirement of only 10 ng DNA for sensitive chimerism analysis may be an advantage of the KMRtrack kit, as demonstrated herein. It is possible that higher doses of DNA would further increase sensitivity of the kit’s qPCR reaction, since various other qPCR-based methods usually need volumes of DNA over 100 ng to obtain higher sensitivity than STR-PCR^8,12^. On the other hand, the KMR kits have also been applied to digital PCR techniques as an alternative to real-time qPCR. Droplet digital PCR with KMRtrack showed a 0.008% detection limit^9^. However, sensitivity of the KMRtrack kit with qPCR as observed in this study is sufficient for its clinical purpose, considering biological background of a normal recipient chimerism level of typically 0.1–0.5% observed in HSCT recipients. Recently, Pedini et al. evaluated chimerism with either qPCR or crystal digital PCR (cdPCR) using KMRtrack. The results of chimerism by qPCR and cdPCR highly correlated with each other. They also found that chimerism results of a next generation sequencing method highly correlated with those of qPCR with KMRtrack^10^. Our data and these findings together indicate the excellent utility of KMR kits for chimerism analysis with various qPCR and digital PCR technologies. For indel based chimerism analysis, including qPCR and digital PCR with KMR markers, as well as next-generation sequencing, use of multiple markers is recommended to increase accuracy of the tests, regarding the examples like chromosomal abnormalities coinciding with the markers’ position in relapses.

In conclusion, chimerism analysis with KMR kits is standardized, sensitive, and highly informative to detect recipient chimerism in allo-HSCT recipients. Therefore, this method could harmonize chimerism analysis for detection of recipient cells and serve as a surrogate marker for graft rejection relapse prediction in hematologic malignancies.

## Supplementary Information

Supplementary Information 1.

Supplementary Information 2.

Supplementary Information 3.

Supplementary Information 4.

Supplementary Information 5.

Supplementary Information 6.

## Data Availability

All data analyzed by the KMR kits during this study are included in this published article and its supplementary information files (Supplemental Tables 6–9). The anonymized clinical data, including formal reports of chimerism analysis evaluated by STR-PCR or in-house SNP-qPCR, used during the current study are available from the corresponding author on reasonable request.
