# Supplementary material for: Evaluation of a quantitative PCR-based method for chimerism analysis of Japanese donor/recipient pairs
Source: Sci Rep. 2022 Dec 9;12:21328. doi: 10.1038/s41598-022-25878-9 (PMC9734659; doi:10.1038/s41598-022-25878-9)
Supplement: Supplementary file 1 — Supplementary Information 1. [file 41598_2022_25878_MOESM1_ESM.pdf]

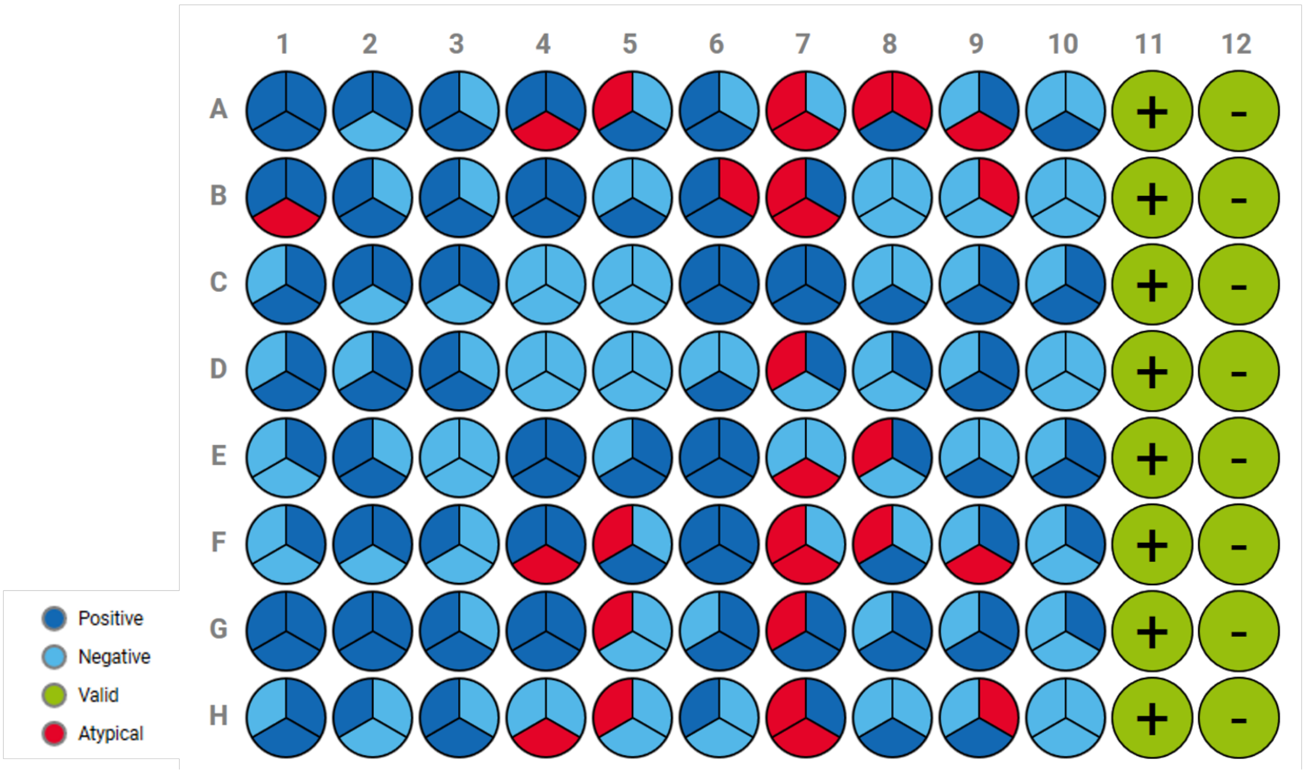

Supplemental Figure 1. Results analyzed by KMRengine for pre-transplant typing with KMRtype Core kit. Letters A-H for rows indicate individuals of donor/recipient pairs, as A/B, C/D, E/F, and G/H. Numbers 1-10 for columns correspond to mixes 1 to 10, described in Supplemental Table 1. Each well shows results of 3 markers for an individual. Dark or light blue results indicate appropriate PCR reactions, whereas red results were atypical because cycle threshold values were out of range. Blue markers with a difference in darkness between donor and recipient are informative for post-transplant chimerism analysis. For example, markers in the bottom of well 2, the right of well 7, the bottom of well 8, and the bottom of well 10 are informative to distinguish A (donor) and B (recipient). The columns with numbers 11 and 12 indicate results of positive and negative controls, respectively, to validate the quality of each sample.

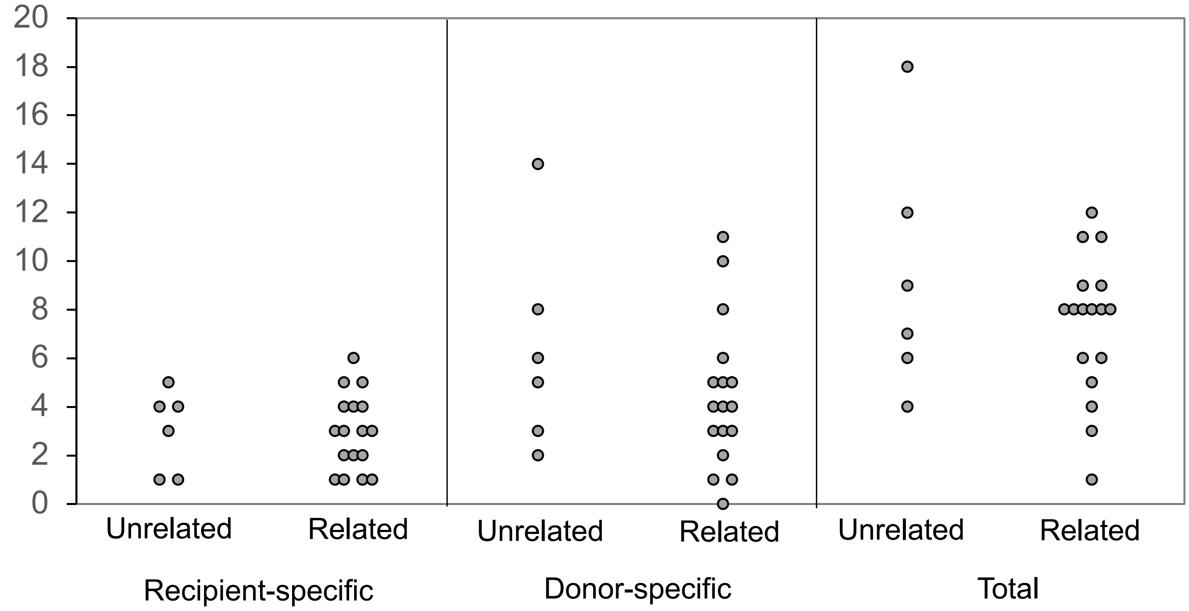

Supplemental Figure 2. Numbers of recipient-specific, donor-specific, and total informative markers in two KMRtype kits for donor/recipient pair with ≤3 recipient-specific markers in the Core kit. We evaluated if KMRtype Extended could add informativity to the 23 of 65 donor/recipient pairs with no more than 3 recipient-specific markers in KMRtype Core, because use of multiple recipient-specific markers is preferable for detection of recipient cells in qPCR-based methods. The other 42 pairs with ≥4 recipient-specific markers in the Core kit are not included in this graph.

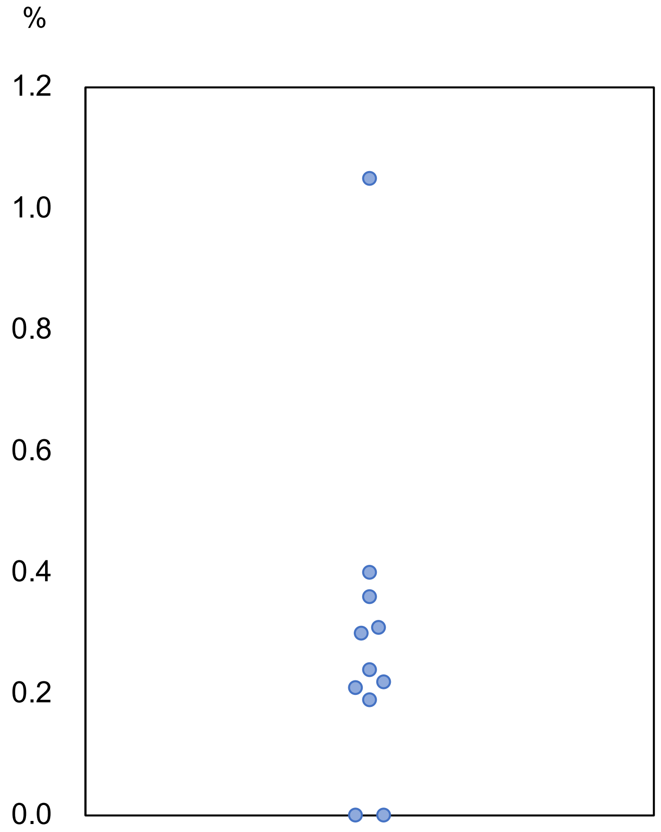

Supplemental Figure 3. Detection of recipient chimerism with KMRtrack in the samples negative for recipient chimerism by STR-PCR. We evaluated 10 samples in which recipient chimerism was negative in STR-PCR, and previously judged as complete donor chimerism. KMRtrack detected minor recipient chimerism in 8 out of these 10 samples. Values of recipient chimerism examined by KMRtrack kit are shown.
