# Supplementary material for: Evaluation of a quantitative PCR-based method for chimerism analysis of Japanese donor/recipient pairs
Source: Sci Rep. 2022 Dec 9;12:21328. doi: 10.1038/s41598-022-25878-9 (PMC9734659; doi:10.1038/s41598-022-25878-9)
Supplement: Supplementary file 2 — Supplementary Information 2. [file 41598_2022_25878_MOESM2_ESM.pdf]

**Supplemental Table 1. Markers in KMRtype kits**

| Kit | Mix | Marker | Chromosome | Informativity |
|---|---|---|---|---|
| KMRtype Core | Mix 01 | KMR050 | 1p | 23/65 (35.4%) |
| | | KMR035 | 2q | 10/65 (15.4%) |
| | | KMR036 | 5q | 19/65 (29.2%) |
| | Mix 02 | KMR037 | 22q | 26/65 (40.0%) |
| | | KMR038 | 5q | 19/65 (29.2%) |
| | | KMR039 | 17p | 23/65 (35.4%) |
| | Mix 03 | KMR040 | 7q | 11/65 (16.9%) |
| | | KMR041 | 5q | 29/65 (44.6%) |
| | | KMR042 | 2q | 14/65 (21.5%) |
| | Mix 04 | KMR043 | 1p | 23/65 (35.4%) |
| | | KMR051 | 4q | 28/65 (43.1%) |
| | | KMR011 | Xp | 20/65 (30.8%) |
| | Mix 05 | KMR009 | 17p | 20/65 (30.8%) |
| | | KMR052 | 4q | 24/65 (36.9%) |
| | | KMR053 | 7p | 11/65 (16.9%) |
| | Mix 06 | KMR044 | Xq | 23/65 (35.4%) |
| | | KMR013 | 6q | 21/65 (32.3%) |
| | | KMR054 | Yp | 18/65 (27.7%) |
| | Mix 07 | KMR055 | 11q | 1/65 (1.5%) |
| | | KMR016 | 17q | 23/65 (35.4%) |
| | | KMR056 | 1p | 16/65 (24.6%) |
| | Mix 08 | KMR045 | 10q | 25/65 (38.5%) |
| | | KMR028 | 20q | 26/65 (40.0%) |
| | | KMR046 | 22q | 14/65 (21.5%) |
| | Mix 09 | KMR047 | 18q | 21/65 (32.3%) |
| | | KMR048 | 14q | 5/65 (7.7%) |
| | | KMR030 | 9q | 16/65 (24.6%) |
| | Mix 10 | KMR057 | 8q | 1/65 (1.5%) |
| | | KMR019 | 20q | 28/65 (43.1%) |
| | | KMR049 | 18q | 28/65 (43.1%) |

| | | | | |
|---|---|---|---|---|
| KMRtype extended | Mix 11 | KMR014 | 12q | 5/23(21.7%) |
| | | KMR034 | 1q | 2/23(8.7%) |
| | | KMR029 | 2q | 5/23(21.7%) |
| | Mix 12 | KMR017 | Yp | 7/23(30.4%) |
| | | KMR004 | 18q | 7/23(30.4%) |
| | | KMR031 | 11p | 2/23(8.7%) |
| | Mix 13 | KMR033 | 12q | 4/23(17.4%) |
| | | KMR010 | 5q | 8/23(34.8%) |
| | | KMR020 | 1p | 6/23(26.1%) |

**Supplemental Table 2. Numbers of informative markers in a total of both KMRtype Core and Extend kits in donor/recipient pairs with ≤3 recipient-specific markers in KMRtype Core kit**

| UID | Related/unrelated | Recipient-specific marker | | | Donor-specific marker | |
|---|---|---|---|---|---|---|
| | | **Core** | **Extended** | **Total** | **Core** | **Extended** |
| 4153* | Unrelated | 1 | 0 | 1 | 2 | 1 |
| 4179 | Related | 2 | 1 | 3 | 3 | 0 |
| 4334 | Related | 2 | 0 | 2 | 6 | 0 |
| 4341 | Related | 3 | 2 | 5 | 3 | 0 |
| 4391 | Unrelated | 3 | 1 | 4 | 6 | 2 |
| 4491* | Related | 1 | 0 | 1 | 9 | 1 |
| 4492 | Related | 2 | 1 | 3 | 7 | 1 |
| 4533* | Unrelated | 0 | 1 | 1 | 3 | 2 |
| 4581 | Related | 2 | 2 | 4 | 3 | 1 |
| 4657* | Related | 1 | 0 | 1 | 0 | 0 |
| 4826 | Related | 1 | 1 | 2 | 1 | 1 |
| 4848 | Related | 2 | 2 | 4 | 4 | 1 |
| 5040 | Related | 3 | 2 | 5 | 1 | 0 |
| 5143 | Related | 2 | 1 | 3 | 4 | 1 |
| 5165* | Related | 1 | 0 | 1 | 1 | 3 |
| 5231 | Related | 2 | 0 | 2 | 1 | 0 |
| 5385* | Related | 1 | 0 | 1 | 9 | 2 |
| 5488 | Unrelated | 3 | 2 | 5 | 2 | 0 |
| 5507 | Related | 2 | 4 | 6 | 2 | 1 |
| 5596 | Unrelated | 3 | 1 | 4 | 11 | 3 |
| 5654 | Related | 2 | 1 | 3 | 4 | 1 |
| 5846 | Unrelated | 2 | 1 | 3 | 6 | 0 |
| 5863 | Related | 3 | 1 | 4 | 4 | 0 |

*: donor/recipient pairs with only one recipient-specific marker.

**Supplemental Table 3. Recipient chimerism in validation experiments with two KMR markers**

| Sample | STR-PCR | KMR marker 1 | | KMR marker 2 | | KMR mean |
|---|---|---|---|---|---|---|
| | | Marker | Value | Marker | Value | |
| 4352 | 0% | KMR041 | 0.19% | KMR052 | 0.46% | 0.32% |
| 4686 | 0% | KMR041 | 0% | KMR048 | 0% | 0% |
| 5387 | 0% | KMR049 | 0.30% | KMR040 | 0% | 0.15% |
| 5403 | 0% | KMR028 | 0.24% | KMR009 | 0% | 0.12% |
| 4652 | 0% | KMR019 | 0.51% | KMR037 | 0.40% | 0.46% |
| 5556 | 0% | KMR045 | 0.21% | KMR049 | 0.32% | 0.27% |
| 4987 | 6% | KMR041 | 1.20% | KMR051 | 1.67% | 1.44% |
| 5125 | 2% | KMR045 | 1.77% | KMR041 | 2.01% | 1.89% |
| 4558 | 18% | KMR052 | 7.44% | KMR037 | 5.46% | 6.45% |
| 4564 | 34% | KMR037 | 45.21% | KMR013 | 32.65% | 38.93% |
| 5803 | 97% | KMR037 | 97.94% | KMR045 | 100% | 98.97% |
| 4544 | 94% | KMR041 | 89.03% | KMR013 | 76.1% | 82.57% |
| 5065 | 70% | KMR037 | 63.35% | KMR038 | 78.66% | 71.01% |

Individual data in Figure 5A-B are shown.

**Supplemental Table 4. Increasing recipient chimerism kinetics in a patient evaluated with 3 KMR markers**

| UID4877 | STR-PCR | KMR045 | KMR050 | KMR051 | KMR mean |
|---|---|---|---|---|---|
| Day27 | 0% | 0.19% | 0.28% | 0.54% | 0.34% |
| Day60 | 0% | 0.13% | 0.35% | 0.23% | 0.24% |
| Day88 | 0% | 0.06% | 0.37% | 0.40% | 0.28% |
| Day116 | 0% | 0.22% | 0.23% | 0.59% | 0.35% |
| Day193 | 38% | 23.15% | 53.69% | 38.48% | 38.44% |
| Day250 | 54% | 40.13% | 60.35% | 57.70% | 52.73% |

Individual data in Figure 5C are shown.

**Supplemental Table 5. Decreasing recipient chimerism kinetics in a patient evaluated with 2 KMR markers**

| UID5083 | STR | KMR041 | KMR045 | KMR mean |
|---|---|---|---|---|
| Day19 | 93% | 100% | - | 100% |
| Day26 | 2% | 2.01% | 1.77% | 1.89% |

Individual data in Figure 5C are shown.
