# Supplementary material for: Evaluation of a quantitative PCR-based method for chimerism analysis of Japanese donor/recipient pairs
Source: Sci Rep. 2022 Dec 9;12:21328. doi: 10.1038/s41598-022-25878-9 (PMC9734659; doi:10.1038/s41598-022-25878-9)
Supplement: Supplementary file 3 — Supplementary Information 3. [file 41598_2022_25878_MOESM3_ESM.pdf]

Supplemental Table 6. Raw mean Cq values evaluated by KMRtype Core kit with KMRengine

UID	Mix1			Mix2			Mix3			Mix4			Mix5			Mix6			Mix7			Mix8			MIx9			Mix10				
	KMR050	KMR035	KMR036	KMR037	KMR038	KMR039	KMR040	KMR041	KMR042	KMR043	KMR051	KMR011	KMR009	KMR052	KMR053	KMR044	KMR013	KMR054	KMR055	KMR016	KMR056	KMR045	KMR028	KMR046	KMR047	KMR048	KMR030	KMR057	KMR049	KMR019	POS	NTC
3521	29.215	29.719	40	40	40	28.473	40	31.764	31.225	27.84	27.078	25.949	40	40	26.303	29.989	28.965	28.561	27.976	29.094	28.425	30.151	40	40	28.932	36.319	40	40	27.852	31.186	29.792	40
3523	40	30.117	29.24	29.538	28.796	28.58	40	40	30.488	28.086	27.271	30.682	40	40	25.687	40	27.942	27.923	26.956	40	27.814	30.306	29.136	40	26.898	40	28.392	40	38.997	30.033	29.042	40
4153	40	31.136	30.427	31.127	29.62	40	40	32.466	31.925	30.825	40	40	40	31.189	29.191	30.502	29.964	30.585	29.298	30.457	34.368	40	40	40	29.575	40	29.342	40	29.688	40	31.256	40
4179	28.295	31.441	29.177	29.397	27.941	28.162	40	30.978	30.077	40	29.369	25.966	40	30.099	40	40	40	29.265	28.054	28.334	26.138	29.918	40	28.049	28.281	40	27.367	35.712	29.588	40	29.795	40
4236	29.152	29.174	28.529	40	40	29.028	40	30.471	30.549	40	29.068	31.447	40	40	27.465	40	28.625	28.519	27.049	28.235	26.756	40	40	40	40	40	40	36.387	40	40	29.921	40
4247	40	40	40	40	28.693	29.881	40	40	31.735	30.294	40	40	40	40	28.604	40	40	29.745	29.792	40	26.994	30.739	40	28.968	29.034	40	40	40	29.913	30.971	30.604	40
4297	28.808	28.671	40	40	28.752	28.7	40	40	28.642	28.816	40	40	40	40	27.388	40	40	40	28.384	28.662	27.483	40	40	40	40	40	40	40	28.136	29.095	28.867	40
4329	40	29.767	29.104	40	40	29.413	40	30.835	29.895	28.473	29.278	31.365	40	29.967	40	40	28.246	29.067	27.936	27.942	31.957	29.893	28.553	40	29.32	40	27.226	40	40	29.494	29.79	40
4332	27.753	31.49	28.091	28.098	28.218	28.638	40	40	31.245	28.885	28.748	30.83	40	40	26.584	28.619	28.214	28.45	27.281	29.192	33.025	29.544	40	27.573	27.807	40	27.18	40	27.789	40	29.355	40
4334	40	28.844	40	40	40	28.789	40	29.889	29.734	29.039	40	40	40	40	40	28.206	28.042	28.214	26.969	28.674	37.005	40	28.151	32.464	28.602	40	26.764	36.265	40	40	29.003	40
4336	28.308	30.741	28.416	40	29.141	29.074	40	30.207	30.203	29.404	40	40	40	29.745	40	28.666	27.375	40	27.523	40	40	40	28.717	32.969	40	40	40	40	40	29.55	29.424	40
4338	30.233	30.053	40	40	40	40	40	31.275	30.519	28.987	40	40	40	30.473	40	29.363	28.544	29.453	28.67	40	40	30.368	28.471	34.266	29.403	29.524	28.036	40	29.109	30.037	30.731	40
4341	30.179	30.422	40	40	30.397	30.273	40	32.228	30.838	30.635	30.578	33.153	40	40	28.758	30.11	29.134	29.968	28.735	40	40	40	29.956	27.015	40	40	40	38.695	40	40	30.775	40
4391	40	33.417	31.442	32.025	30.881	40	40	40	31.932	40	40	40	40	32.322	29.195	31.06	30.283	31.006	30.719	31.756	35.888	40	40	30.498	40	40	29.942	40	40	40	32.309	40
4408	40	30.68	40	40	40	40	40	31.676	40	40	40	30.354	40	40	40	40	28.131	28.725	28.247	29.433	33.99	30.024	29.244	40	29.391	40	28.386	40	40	30.092	29.721	40
4422	29.473	30.879	40	40	40	40	40	40	30.732	40	40	30.36	40	30.208	27.956	28.84	40	28.553	29.303	29.485	34.517	40	40	40	40	40	40	40	29.335	40	29.676	40
4451	40	30.58	27.592	28.354	40	40	40	31.187	30.337	40	40	29.961	40	40	27.496	28.623	40	28.313	27.823	29.09	26.786	40	28.99	40	40	40	27.2	40	27.968	40	29.092	40
4452	28.698	30.798	28.353	30.046	29.382	28.621	40	31.189	30.817	29.474	29.396	32.269	40	40	27.719	29.195	28.136	40	28.102	29.45	27.014	29.988	28.63	40	28.15	40	28.385	40	40	30.103	29.91	40
4459	40	31.308	29.948	40	40	30.931	40	30.76	30.069	29.091	40	40	40	40	28.477	40	40	30.023	28.955	29.677	36.383	30.735	40	40	29.873	40	29.095	40	40	40	31.304	40
4479	29.224	29.735	27.806	40	40	29.062	40	40	30.675	28.379	40	40	28.38	28.942	27.713	28.732	27.996	40	28.028	28.964	33.196	40	28.479	40	40	40	40	40	29.245	40	29.626	40
4491	30.563	31.19	28.904	30.936	40	40	40	40	30.281	30.788	40	31.666	29.445	40	40	40	28.96	40	29.955	40	40	40	40	40	40	40	40	40	40	40	31.237	40
4492	28.777	40	28.555	29.08	28.433	28.503	40	40	30.409	40	28.903	25.829	28.667	40	27.371	27.687	40	40	27.179	40	40	40	40	40	27.678	40	27.976	40	40	40	29.105	40
4493	29.288	30.676	29.69	30.491	30.012	29.113	40	31.959	30.466	40	30.485	33.106	40	40	40	29.954	40	29.85	28.901	30.296	37.398	40	29.755	34.243	30.113	40	28.324	40	29.731	40	30.595	40
4501	40	29.805	29.346	29.69	40	40	40	40	29.884	40	40	40	28.648	40	40	29.016	27.38	40	27.994	28.406	27.057	40	40	40	29.355	40	28.337	40	29.135	30.158	29.839	40
4512	29.542	29.853	29.173	30.136	28.985	29.492	40	40	30.697	40	40	30.328	29.04	40	28.122	29.459	28.317	28.92	28.866	40	40	30.285	40	40	29.461	40	28.66	40	29.366	30.362	30.502	40
4533	40	29.441	28.769	29.786	28.314	29.021	40	31.25	30.006	29.224	40	26.196	40	28.788	40	40	28.971	25.735	28.977	29.283	38.766	40	40	40	40	40	28.857	40	40	29.454	29.719	40
4547	28.302	31.226	29.062	40	28.846	28.172	40	40	29.982	29.446	29.406	40	40	40	28.083	29.191	28.151	28.772	28.571	28.649	33.632	30.056	40	40	28.424	40	28.388	40	40	40	29.853	40
4562	40	31.116	28.476	40	40	40	40	40	31.102	28.385	29.158	31.679	40	40	27.885	29.268	28.684	40	28.082	40	40	40	40	28.263	40	40	40	40	29.28	40	30.095	40
4572	30.991	31.417	29.99	40	34.431	40	37.412	36.73	30.799	30.274	40	40	40	28.339	38.916	30.131	29.215	30.336	28.544	32.26	33.063	30.101	29.335	40	30.038	38.802	32.427	33.16	34.827	34.686	31.646	40
4574	28.96	28.459	28.971	40	28.156	34.336	40	40	30.219	29.137	40	40	28.402	27.234	37.045	28.408	37.503	40	26.89	40	40	28.798	28.582	27.317	27.966	36.184	40	38.635	40	40	30.093	40
4581	31.286	40	30.355	32.042	29.589	35.097	40	32.338	31.387	31.576	34.751	38.691	29.438	32.691	40	29.754	37.56	40	30.055	28.966	33.877	31.509	29.988	40	30.141	36.654	28.973	35.577	30.904	34.867	32.329	40
4583	31.036	40	29.722	31.739	29.35	26.969	36.273	40	30.179	30.239	28.63	32.697	29.391	31.953	28.265	29.885	40	30.028	28.957	28.168	34.009	30.611	28.784	28.846	29.833	40	28.573	34.168	29.274	28.09	31.785	40
4586	29.108	29.166	29.018	40	28.379	29.125	40	31.299	40	40	29.628	31.233	40	30.481	40	28.99	27.535	28.89	27.828	40	40	29.057	40	40	29.083	40	28.494	40	29.1	40	29.799	40
4602	28.965	29.418	28.998	40	40	29.633	30.5	30.696	40	29.03	28.206	31.566	29.362	40	26.195	28.932	28.819	30.173	28.277	40	40	40	40	40	27.715	40	28.254	36	36.667	28.909	29.775	40
4603	40	28.768	40	29.567	40	40	29.87	40	29.135	28.372	40	25.629	28.432	29.426	26.51	28.358	40	28.005	27.862	40	40	40	40	40	27.054	40	27.738	40	35.794	27.996	28.87	40
4623	40	30.843	28.425	28.669	28.852	40	40	40	31.078	29.416	40	40	40	29.984	40	29.25	28.367	28.967	27.729	29.476	27.067	28.955	28.981	40	29.339	40	27.692	40	28.357	40	29.902	40
4657	29.247	29.995	27.593	29.172	26.853	26.426	29.622	40	40	29.396	40	40	27.898	29.038	26.63	28.9	28.251	28.496	27.921	28.557	33.225	29.113	28.052	25.525	27.936	36.696	40	40	27.713	40	29.44	40
4659	29.306	29.902	40	29.281	26.899	26.52	29.764	40	40	28.441	40	40	28.283	29.049	25.776	28.953	27.277	28.347	28.607	28.529	33.286	29.229	28.175	33.328	27.704	38.613	40	40	27.496	40	29.447	40
4705	40	29.322	28.975	40	40	29.186	30.218	29.962	30.177	29.532	29.32	31.835	40	40	28.194	29.03	40	40	28.037	28.215	27.945	28.387	40	40	40	40	28.839	40	29.249	40	29.977	40
4733	40	29.092	28.951	29.301	40	40	40	40	30.543	29.478	40	40	28.711	40	40	40	40	28.716	27.466	40	40	40	28.255	32.514	29.093	40	28.544	38.397	40	40	29.598	40
4762	40	31.484	40	30.109	40	29.54	31.037	40	30.392	30.393	30.325	32.185	29.602	40	40	29.798	28.859	29.73	29.605	30.181	28.928	29.152	29.14	33.346	28.917	40	28.262	35.734	40	30.333	30.83	40
4763	40	27.704	40	26.999	40	27.778	40	29.715	29.153	27.955	40	24.517	40	28.909	27.443	40	26.25	27.643	26.464	26.803	25.383	28.618	27.6	31.177	26.799	40	27.024	40	27.565	28.221	28.192	40
4772	40	31.918	30.142	31.165	29.358	29.404	40	40	31.789	30.376	30.338	32.814	40	40	40	30.252	30.172	30.037	30.295	30.522	38.373	29.655	40	27.735	30.159	40	29.195	40	30.621	40	32.359	40
4785	40	30.408	29.168	40	40	30.073	40	31.355	30.461	30.342	30.417	32.432	40	40	29.713	40	29.871	30.003	29.05	29.043	33.054	40	29.175	32.846	30.013	38.837	40	40	40	40	30.82	40
4805	29.244	29.568	29.232	28.594	40	40	30.177	40	30.794	29.606	40	40	29.785	40	40	40	28.772	29.022	28.002	40	40	40	40	40	28.118	40	27.608	40	40	29.96	29.746	40
4816	29.906	29.943	28.702	40	30.134	40	40	40	30.006	40	29.188	30.538	40	30.538	40	40	40	28.575	28.932	28.75	28.454	29.948	40	28.479	40	40	29.473	40	29.585	29.413	30.005	40
4826	40	31.441	40	30.657	40	30.247	40	31.748	30.535	30.017	40	40	40	40	28.443	28.177	28.644	32.759	27.911	29.965	27.304	29.931	28.531	27.752	40	40	29.141	39.47	40	40	30.588	40
4839	29.292	40	28.84	40	29.375	40	40	40	30.256	29.961	40	40	40	29.491	40	27.974	40	40	27.976	29.023	38.41	29.135	40	40	28.176	40	40	40	28.49	29.393	29.26	40
4848	29.971	30.65	29.286	30.563	28.686	25.931	40	29.36	30.989	30.286	40	40	28.498	40	28.297	40	40	29.523	28.843	27.945	33.344	30.149	40	40	30.007	37.601	40	40	40	40	30.619	40
4850	28.443	30.597	28.951	40	28.413	25.544	37.648	29.985	40	29.745	40	40	27.108	34.577	40	29.161	28.065	28.904	28.01	40	27.336	29.645	40	27.41	29.479	36.912	40	40	28.91	40	30.044	40
4877	29.888	33.775	28.799	30.924	33.409	32.659	40	30.92	30.534	40	30.779	31.461	40	40	32.785	40	40	40	29.738	33.798	27.559	29.513	40	32.807	30.133	28.647	29.446	40	40	29.487	31.264	40
4878	40	31.507	28.674	40	40	27.184	40	40	31.322	40	40	40	40	30.096	36.042	29.478	40	40	27.512	29.316	27.379	40	40	40	40	40	28.345	37.555	40	40	29.971	40
4890	29.97	31.01	29.772	40	29.66	40	30.489	40	30.205	29.762	40	40	29.141	29.395	40	40	28.808	40	28.77	29.081	34.719	40	28.969	33.124	29.404	40	29.138	40	40	40	29.873	40
4898	29.967	29.283	40	40	29.319	28.067	40	29.989	28.978	29.739	29.573	31.939	40	29.991	40	29.294	28.299	28.929	28.547	29.41	27.408	40	28.571	33.112	40	40	29.171	40	40	29.76	30.078	40
4899	40	30.529	29.048	40	29.291	29.394	40	30.567	29.14	40	28.93	31.244	28.53	40	26.659	28.232	27.719	25.46	28.735	29.155	26.491	40	40	27.275	40	30.27	28.15	38.923	28.938	29.748	29.902	40
4914	40	28.99	29.43	29.316	27.94	28.769	40	30.63	30.112	27.789	40	40	40	28.543	40	28.961	28.473	33.217	28.588	28.132	36.379	40	40	27.735	40	40	28.601	40	28.27	29.064	29.26	40
4922	40	40	40	40	40	40	40	40	30.694	40	40	25.803	28.814	40	28.042	28.955	40	28.575	27.632	40	40	40	40	25.622	29.144	40	28.651	40	29.264	40	30.253	40
4936	29.95	30.994	29.712	29.411	29.35	28.534	40	30.958	30.911	40	40	40	40	40	40	29.677	40	28.224	28.479	29.682	40	29.994	40	40	28.777	40	28.963	40	40	30.218	30.127	40
4941	40	31.135	40	30.549	40	40	40	40	40	40	40	40	40	40	30.013	30.211	29.35	29.967	28.79	29.179	32.581	31.185	28.88	32.675	30.458	28.536	29.671	40	40	30.88	30.863	40
4949	40	29.976	40	40	29.018	29.147	40	40	30.011	28.85	40	40	40	29.567	40	40	40	25.321	26.932	40	40	40	40	40	28.737	29.214	27.531	40	27.653	29.393	29.33	40
4950	40	31.943	28.229	40	29.284	40	39.283	40	40	40	40	40	40	29.889	27.653	40	29.251	29.486	27.765	29.116	27.669	29.988	28.874	33.322	40	40	29.297	40	28.534	40	30.431	40
4952	39.553	32.601	28.747	40	29.945	40	40	40	31.379	37.33	40	40	34.945	40	40	30.09	28.84	29.997	29.506	28.732	32.126	30.696	40	40	30.121	40	29.75	37.096	33.881	34.514	31.05	40
4963	40	40	30.193	30.452	29.976	40	40	40	30.289	29.888	40	40	40	40	27.384	28.345	28.673	32.979	28.961	30.151	28.171	40	28.699	40	28.797	40	28.828	40	40	40	30.429	40
4983	40	30.422	40	31.214	30.19	29.157	40	40	30.782	40	30.09	26.92	40	40	28.721	28.816	29.236	40	29.973	29.928	37.144	30.171	40	27.023	29.436	40	28.917	40	28.739	30.256	31.517	40
5016	40	40	28.513	29.965	28.806	27.971	40	31.891	30.855	29.862	40	40	40	40	40	29.486	28.973	40	27.977	29.705	27.84	40	40	40	29.198	40	28.984	40	28.802	30.174	29.607	40
5040	29.487	30.127	28.508	40	40	40	40	31.628	40	29.619	29.688	31.996	29.436	40	28.078	28.968	40	40	28.434	40	27.512	40	40	40	29.566	36.663	40	40	29.075	29.991	30.502	36.762
5043	39.436	31.606	34.92	30.954	29.744	35.31	31.95	40	30.383	31.414	31.146	40	40	40	28.563	40	30.429	30.668	29.322	29.912	33.944	40	29.904	27.807	40	40	40	38.113	29.784	38.711	31.56	40
5044	31.392	32.76	40	32.205	38.034	40	31.416	31.948	30.256	40	40	40	40	40	27.614	40	30.009	30.568	29.44	28.558	33.68	40	40	40	29.853	40	30.114	40	29.161	40	31.954	40
5046	28.971	29.009	40	29.369	40	28.994	40	40	30.659	40	29.404	31.362	40	40	27.694	28.992	40	40	27.754	40	40	40	40	40	29.042	40	28.605	40	28.802	29.875	29.628	40
5058	40	32.166	40	40	40	30.911	40	31.878	30.658	30.308	40	40	40	40	29.177	29.288	29.148	26.63	28.935	29.837	28.931	30.062	28.537	28.344	29.704	40	29.132	35.925	40	40	31.277	40
5083	39.307	40	40	31.294	31.352	31.315	32.768	34.965	32.933	40	32.127	32.814	40	40	29.986	30.666	40	40	30.86	30.733	38.503	31.939	31.338	40	31.678	40	31.063	40	31.291	32.531	32.134	40
5095	40	29.865	40	40	40	28.816	40	30.824	30.079	40	28.71	30.848	40	40	26.58	28.864	28.463	32.65	27.059	29.114	27.578	29.065	40	27.221	28.627	40	27.323	40	28.559	40	29.168	40
5098	40	29.213	29.143	40	40	29.25	40	40	40	29.102	29.249	31.663	40	40	27.977	29.542	40	40	28.052	28.597	37.093	29.691	28.087	27.669	28.218	40	27.796	40	28.07	40	29.676	40
5099	40	30.088	40	29.503	27.744	28.829	40	40	29.447	29.889	40	40	28.663	29.66	40	29.09	28.849	27.618	27.964	29.225	27.859	40	27.309	40	40	40	40	40	27.73	29.645	29.183	40
5103	29.182	30.617	28.781	28.4	40	40	40	40	29.47	28.253	40	40	40	29.878	40	28.732	27.94	28.534	27.681	29.018	27.553	28.281	40	25.765	28.909	40	40	40	28.707	29.608	29.553	40
5137	40	30.287	40	40	40	29.101	40	40	30.107	28.894	40	40	40	29.611	27.598	40	28.695	32.404	28.723	29.06	27.681	29.526	27.98	40	40	40	40	40	40	29.436	29.397	40
5141	40	28.121	40	29.683	27.851	34.129	40	29.927	29.746	28.095	40	40	40	36.467	27.309	40	26.735	28.567	26.53	27.45	25.904	29.389	27.906	27.054	28.042	40	26.983	37.531	40	27.332	29.878	40
5143	40	32.653	29.793	40	31.2	30.995	40	32.84	40	28.564	40	27.584	40	29.874	29.278	40	28.456	40	27.917	30.018	34.207	29.933	40	40	28.234	40	30.074	40	40	40	30.624	40
5148	40	30.832	28.09	40	29.596	40	36.666	30.887	40	27.425	27.89	30.42	40	28.218	40	40	26.687	28.119	25.621	29.124	26.552	27.891	40	40	27.318	29.312	28.152	40	35.314	29.869	28.652	40
5149	40	32.408	40	40	40	40	40	40	29.681	28.524	29.24	26.363	40	40	40	28.97	40	36.756	28.205	29.612	35.621	29.587	28.029	40	29.261	40	27.417	40	40	40	30.437	40
5162	40	29.329	28.11	40	40	40	36.432	40	40	40	29.207	31.29	28.851	40	27.705	29.362	40	40	27.297	40	27.938	40	28.208	40	28.014	33.708	40	40	27.685	29.711	29.569	40
5165	40	32.315	40	31.331	30.189	36.992	40	32.73	32.997	31.945	40	40	40	31.287	28.803	40	30.917	31.071	30.024	40	29.563	31.106	40	29.074	30.732	40	29.013	35.945	31.122	40	32.378	40
5177	40	32.137	30.453	40	31.12	40	40	40	31.518	30.441	30.677	32.962	40	40	29.195	40	29.567	33.769	30.578	30.712	39.873	30.986	29.459	29.043	40	40	30.543	40	40	30.958	31.323	40
5187	29.657	29.276	40	30.09	29.114	29.321	40	40	30.635	30.149	40	40	40	40	27.792	29.471	40	35.246	28.129	29.579	28.113	40	40	28.267	29.501	40	28.939	40	28.843	29.105	30.345	40
5231	29.133	28.773	40	29.518	28.662	40	40	40	30.285	29.747	40	40	40	29.478	27.168	28.93	28.771	27.37	27.988	29.07	27.662	40	40	27.884	27.879	40	27.477	40	28.265	28.408	29.974	40
5246	40	30.109	40	40	30.566	29.657	40	40	40	29.367	29.343	31.971	29.981	30.965	40	30.23	29.017	29.896	28.743	40	40	40	29.83	28.959	29.393	38.234	40	40	30.045	31.227	30.975	40
5297	34.234	31.225	27.606	40	40	29.937	40	40	30.173	34.394	28.812	31.387	40	34.984	28.008	40	27.476	40	26.206	28.685	27.092	40	28.356	31.782	27.714	40	28.066	40	40	40	28.935	40
5298	40	30.796	28.981	40	40	40	40	40	29.606	40	28.44	31.163	40	40	27.868	40	40	28.65	26.166	40	40	40	40	40	40	40	28.277	40	27.142	30.086	28.697	40
5308	40	31.018	30.191	30.787	40	30.297	31.281	40	30.675	40	40	40	40	40	40	29.959	28.968	29.779	28.587	29.452	28.497	30.722	40	40	29.959	40	29.136	40	29.789	30.237	30.477	40
5309	40	31.736	30.1	30.948	40	40	31.439	40	30.342	30.147	29.92	32.044	29.689	40	28.846	28.545	40	35.536	29.421	29.183	33.695	30.315	40	28.486	30.173	40	29.201	40	30.105	40	30.931	40
5347	30.629	33.492	40	32.358	40	40	40	40	40	40	30.907	28.199	40	30.183	29.198	40	29.969	30.954	28.242	40	29.407	30.951	40	28.716	29.964	40	30.428	40	38.355	31.706	31.391	40
5348	40	30.815	40	40	40	29.373	40	40	30.792	40	28.126	25.548	40	40	27.696	28.508	26.275	28.403	25.409	28.708	32.821	28.836	40	40	40	40	40	40	27.207	40	28.737	40
5354	29.057	31.895	28.975	28.146	40	40	40	40	40	28.39	40	40	40	40	26.47	27.162	27.785	32.4	26.654	40	27.063	28.801	27.313	26.667	28.738	40	28.077	40	40	29.857	29.397	40
5359	40	30.609	40	30.297	40	29.94	40	40	30.964	28.351	29.254	32.396	40	28.991	27.783	29.325	28.534	29.075	28.076	40	27.975	30.174	40	28.288	28.306	40	28.533	40	40	30.09	30.378	40
5362	29.846	31.607	28.468	30.306	29.849	29.052	40	40	30.245	30.078	29.901	32.116	40	30.716	28.536	29.385	40	29.472	28.202	40	28.285	40	40	35.24	28.532	40	28.677	40	29.48	40	30.076	40
5376	28.407	31.722	29.028	40	40	40	40	30.038	30.524	27.256	28.538	31.52	40	40	28.063	28.891	27.615	28.942	26.609	29.061	33.12	40	28.812	26.985	40	28.321	40	40	26.427	40	29.237	40
5385	31.895	40	40	40	38.307	40	40	40	31.374	30.075	30.055	35.07	40	40	40	29.933	40	40	34.282	32.169	40	40	35.264	29.751	31.244	40	40	40	31.335	40	31.212	40
5422	40	28.647	28.378	29.382	27.971	40	40	30.973	30.915	40	40	40	40	40	40	28.09	40	40	40	29.518	35.386	40	27.751	40	27.667	40	28.249	40	40	40	29.106	40
5428	30.501	32.262	28.99	40	31.36	29.762	40	40	30.44	30.358	29.383	32.724	29.834	40	28.028	30.488	29.868	30.004	29.215	40	28.266	40	30.076	28.718	40	40	29.534	40	29.159	30.616	30.644	40
5429	40	28.992	28.334	40	29.218	28.676	40	30.757	29.386	28.262	27.496	30.838	27.99	40	25.686	40	28.024	28.048	27.387	40	26.866	40	28.9	27.245	40	40	28.867	35.221	40	40	29.218	40
5441	30.036	31.682	29.966	40	30.107	29.922	40	40	40	40	30.228	31.984	40	40	40	29.934	40	29.739	28.743	29.121	32.542	30.963	30.094	33.122	40	40	28.632	40	29.818	40	30.383	40
5488	29.859	30.689	29.235	30.528	40	29.971	40	31.235	30.706	28.33	29.362	32.473	40	29.824	27.888	29.397	40	29.123	28.213	40	28.105	30.031	27.912	28.149	28.258	40	28.9	40	29.335	40	30.379	40
5502	28.887	27.997	27.47	40	28.914	28.777	40	30.138	30.004	28.412	27.508	30.207	40	28.96	39.467	28.491	27.358	28.122	26.788	27.704	26.868	40	40	40	40	40	26.785	40	40	40	29.501	40
5507	40	27.01	25.638	26.677	40	30.332	40	27.876	25.466	40	24.782	22.68	40	26.788	27.377	24.55	25.478	29.262	24.726	28.619	40	40	30.133	24.114	25.005	40	24.828	40	27.832	26.2	25.729	40
5508	40	30.622	27.978	40	40	27.77	40	29.132	30.535	40	28.724	31.014	28.846	40	28.247	28.922	26.883	27.152	27.088	28.315	36.805	29.612	28.111	25.424	28.747	40	27.844	40	27.261	28.718	29.288	40
5509	40	30.408	28.58	40	27.951	28.885	40	40	30.286	27.729	28.38	30.939	28.758	29.384	40	40	28.891	28.985	28.611	29.015	27.095	40	28.43	31.849	28.099	40	27.673	40	40	40	29.224	40
5542	40	40	29.521	40	30.71	40	40	40	30.029	29.726	40	40	40	40	29.946	29.584	29.272	29.546	29.113	40	28.829	30.314	27.779	34.152	29.324	40	28.406	40	29.363	40	31.176	40
5577	28.719	40	28.054	29.141	28.824	28.751	29.541	40	40	28.915	29.003	30.782	40	40	40	28.955	40	28.666	27.308	40	40	40	29.051	25.291	28.863	36.619	40	40	27.797	40	29.541	40
5584	40	32.527	39.406	40	30.428	40	31.93	31.822	30.562	30.969	29.595	31.92	40	38.255	39.862	30.795	29.821	37.487	28.726	29.465	29.4	38.17	29.471	33.402	40	40	29.424	40	28.931	29.199	31.261	40
5585	40	30.669	29.096	40	29.197	40	29.931	29.571	29.59	40	28.76	31.151	40	40	40	40	40	27.376	27.312	40	27.985	40	27.33	31.473	29.009	35.402	40	40	27.304	28.891	29.567	40
5592	40	30.127	25.998	30.34	29.224	28.412	30.51	40	30.381	29.026	29.562	40	40	29.997	29.236	29.405	40	29.494	30.039	40	40	30.164	40	27.982	29.6	40	28.675	40	29.32	29.973	30.216	40
5596	29.977	29.961	29.291	40	40	30.307	40	40	40	39.389	40	40	40	30.6	40	28.532	28.688	40	28.521	40	40	30.235	40	40	28.785	36.953	40	40	29.575	30.461	30.357	40
5597	28.956	29.061	40	29.207	40	29.036	40	31.184	30.795	28.993	40	40	40	40	28.565	28.818	28.087	28.303	27.394	29.302	27.364	29.359	27.984	25.62	28.56	28.14	28.134	40	40	40	29.458	40
5608	32.109	32.423	31.295	40	40	40	40	32.214	37.616	40	40	40	32.043	40	31.029	31.885	30.027	33.872	31.174	40	31.776	32.695	31.19	35.244	32.089	34.897	40	40	34.473	40	32.853	40
5609	29.762	31.676	29.889	40	29.894	40	40	29.204	31.154	29.847	40	40	40	40	40	40	28.736	27.98	28.446	29.28	35.356	29.393	40	40	29.671	37.89	40	40	40	40	30.466	40
5636	29.077	29.257	29.06	28.456	28.673	40	40	31.578	30.334	28.983	28.962	31.304	40	40	28.816	29.171	28.617	40	27.913	40	27.96	29.665	28.369	34.5	40	40	28.887	40	27.628	30.304	28.878	40
5649	29.161	29.328	40	29.461	40	40	40	31.299	30.885	40	40	25.884	40	40	40	40	40	28.793	28.736	29.327	27.76	29.584	27.787	33.456	40	40	27.577	40	28.847	40	29.553	38.559
5653	30.96	32.461	30.11	40	31.362	30.384	40	31.043	31.024	40	29.937	33.187	40	31.298	29.939	40	40	29.233	29.652	29.635	40	40	29.546	34.735	28.898	28.768	40	40	28.659	40	32.577	40
5654	29.633	31.477	28.715	29.962	40	40	40	40	29.544	40	29.763	31.933	40	40	40	40	27.338	29.055	28.225	28.336	27.911	40	40	40	40	40	28.75	40	29.223	40	29.874	40
5657	30.835	33.269	29.231	40	31.074	34.944	40	30.974	31.289	40	36.389	38.62	40	31.604	30.175	40	29.412	34.369	30.228	29.652	29.678	31.208	29.635	34.114	30.663	36.336	40	40	27.995	30.066	31.235	40
5658	40	29.798	25.513	28.725	27.876	29.005	40	40	31.274	40	40	30.494	29.125	40	27.973	28.227	28.911	40	29.41	30.051	33.441	40	40	40	28.375	35.859	40	40	29.103	30.326	30.024	40
5671	30.31	32.422	26.025	30.31	40	29.671	40	30.069	30.66	40	40	40	40	30.167	29.445	40	30.352	40	29.826	28.689	31.687	30.328	40	28.324	28.812	40	28.784	40	29.904	40	30.547	40
5745	40	29.762	40	40	40	28.832	40	40	40	28.689	40	40	40	40	40	40	28.684	28.647	28.531	28.042	31.056	40	28.318	31.798	40	40	27.877	40	28.343	29.105	28.968	40
5754	29.817	30.017	34.002	40	40	40	40	30.379	30.134	29.668	40	40	29.34	40	29.032	40	40	29.508	28.41	29.978	33.317	30.099	28.006	40	29.494	40	28.833	40	28.253	40	29.95	40
5779	40	30.053	33.574	40	27.4	40	40	40	40	28.716	28.443	40	27.991	40	28.215	28.192	28.842	40	27.74	40	40	40	40	40	27.676	40	27.62	40	40	40	29.011	40
5817	40	30.547	34.162	40	29.263	40	40	31.453	29.595	40	40	30.269	40	40	28.971	40	29.884	40	28.985	40	40	30.071	40	40	29.199	40	27.801	40	28.022	30.096	29.824	40
5846	40	30.827	28.933	30.238	40	40	40	40	30.865	29.465	40	25.528	40	40	28.958	29.301	40	40	28.096	28.384	31.386	40	28.855	32.315	29.647	40	28.281	40	40	40	29.651	40
5863	40	30.179	33.756	28.111	28.016	28.423	40	40	40	40	40	29.704	40	40	40	28.471	40	28.687	27.633	27.745	30.85	29.237	26.977	26.925	28.612	40	27.807	40	40	40	29.226	40
5864	28.926	30.439	34.546	40	28.343	28.371	40	40	29.734	28.902	40	29.573	40	28.192	40	40	40	28.626	27.507	27.533	30.812	40	27.03	27.047	28.577	40	27.729	40	40	40	28.985	40
5901	40	29.789	40	29.485	28.391	28.657	40	30.837	29.609	40	40	40	40	40	28.341	28.923	28.693	40	28.101	28.27	26.701	40	28.783	24.897	28.785	40	28.005	40	27.968	29.935	28.933	40
