# Supplementary material for: Evaluation of a quantitative PCR-based method for chimerism analysis of Japanese donor/recipient pairs
Source: Sci Rep. 2022 Dec 9;12:21328. doi: 10.1038/s41598-022-25878-9 (PMC9734659; doi:10.1038/s41598-022-25878-9)
Supplement: Supplementary file 4 — Supplementary Information 4. [file 41598_2022_25878_MOESM4_ESM.pdf]

Supplemental Table 7. Raw mean Cq values evaluated by KMRtype Extended kit with KMRengine

UID	Mix1			Mix2			Mix3				
	KMR014	KMR034	KMR029	KMR017	KMR004	KMR031	KMR033	KMR010	KMR020	POS	NTC
4153	40	40	28.428	26.992	29.275	29.146	40	28.353	34.707	29.186	40
4179	40	28.547	27.46	28.376	29.027	30.495	27.266	27.582	29.637	29.64	40
4332	40	27.194	29.224	28.201	29.731	30.075	27.916	27.367	40	29.464	40
4334	40	26.582	31.412	26.896	40	29.025	28.071	28.01	35.851	29.103	40
4336	40	27.421	35.228	40	29.333	28.674	40	28.305	31.692	28.374	36.952
4338	40	26.655	31.455	26.942	40	28.894	26.757	27.742	40	28.604	40
4341	40	27.666	35.298	28.916	30.797	30.203	29.591	29.633	32.633	30.066	40
4391	40	40	30.805	30.04	40	31.072	30.603	40	40	30.523	40
4491	40	40	40	40	40	40	29.295	29.162	34.689	30.975	40
4492	40	40	40	40	40	28.854	27.811	27.693	26.951	28.814	40
4493	40	27.967	28.772	28.051	40	30.49	28.293	40	28.393	30.191	40
4512	40	40	28.317	40	40	40	27.725	26.769	31.504	29.369	40
4533	27.025	25.588	26.877	28.367	40	29.114	26.253	40	40	28.685	40
4547	28.935	27.868	40	28.076	40	29.142	27.789	28.679	32.038	28.772	40
4581	40	30.397	31.747	40	32.081	31.528	35.386	35.657	40	31.665	40
4583	40	29.192	31.437	29.071	40	30.239	28.984	40	40	30.437	40
4623	40	40	24.996	23.915	25.71	25.567	40	24.71	31.491	25.932	40
4657	40	40	26.119	28.419	40	29.095	27.358	27.511	32.635	28.7	40
4659	40	40	27.327	28.268	40	28.958	26.122	26.334	31.249	28.607	40
4826	40	27.974	29.021	40	40	32.078	28.903	29.88	36.652	29.799	40
4848	40	28.724	34.335	29.304	40	31.593	30.21	30.244	29.5	31.166	40
4850	40	27.613	33.142	27.741	29.784	29.751	28.904	40	40	29.639	40
4914	28.358	24.759	35.708	40	40	29.484	26.777	27.971	33.23	29.028	40
5040	40	27.868	28.576	40	40	29.07	28.512	28.481	31.813	29.393	40
5058	40	26.223	27.17	29.082	40	29.711	28.087	39.153	40	29.77	40
5141	40	22.325	27.811	24.982	40	25.593	23.87	23.922	28.635	25.554	40
5143	40	29.107	29.896	40	40	31.477	30.38	29.079	36.289	31.487	40
5148	40	26.687	32.117	26.899	40	28.698	27.866	26.775	34.356	28.823	40
5162	40	40	40	40	40	28.87	28.649	28.441	31.802	28.819	40
5165	40	40	40	27.947	40	28.701	26.989	40	40	28.536	40
5187	40	40	27.994	40	40	40	40	28.05	33.579	30.968	40
5231	40	40	27.52	40	40	40	27.268	27.473	32.332	29.384	40
5376	29.207	40	28.496	40	40	40	27.745	26.745	31.918	29.118	40
5385	40	40	31.779	40	40	40	31.224	31.166	40	32.967	40
5488	4	26.59	28.183	27.886	40	29.068	28.877	27.911	31.564	29.269	40
5502	40	40	40	27.471	29.177	29.541	40	28.155	31.334	28.762	40
5507	40	27.83	33.254	30.794	32.677	30.943	30.863	28.719	36.05	29.894	40
5508	28.491	25.868	27.42	40	40	40	40	27.498	32.208	28.5577	40
5596	40	40	29.376	40	40	31.109	40	40	40	29.904	40
5636	40	26.623	28.056	40	29.803	40	40	28.245	31.824	28.636	36.446
5649	28.583	40	27.872	27.335	40	30.312	28.414	40	40	29.184	40
5654	40	40	40	27.5	29.721	30.443	28.674	40	40	29.382	40
5846	40	40	28.311	40	40	29.504	28.814	28.599	35.972	29.581	40
5863	40	40	40	27.257	29.029	28.285	28.126	28.173	31.947	28.352	40
5864	40	40	40	27.702	40	29.711	28.364	28.238	31.556	28.584	40
5901	40	26.449	31.742	40	40	28.796	40	26.631	33.313	28.687	40
