# Supplementary material for: Evaluation of a quantitative PCR-based method for chimerism analysis of Japanese donor/recipient pairs
Source: Sci Rep. 2022 Dec 9;12:21328. doi: 10.1038/s41598-022-25878-9 (PMC9734659; doi:10.1038/s41598-022-25878-9)
Supplement: Supplementary file 5 — Supplementary Information 5. [file 41598_2022_25878_MOESM5_ESM.pdf]

Supplemental Table 8. Raw mean Cq values and recipient chimerism evaluated by KMRtrack kit with KMRengine for virtual samples

No.	KMR														
	Marker	Recipient %	Simulated recipient %	Reference Sample						Monitoring Sample					
				Informative marker mean Cq	Informative marker Cq SD	Informative marker Cq CV	Reference assay mean Cq	Reference assay Cq SD	Reference assay Cq CV	Informative marker mean Cq	Informative marker Cq SD	Informative marker Cq CV	Reference assay mean Cq	Reference assay Cq SD	Reference assay Cq CV
1	KMR019	0	0	28.87	0.08	0.26	29.69	0.03	0.11	40.00	0.00	0.00	29.10	0.16	0.56
2	KMR019	0	0	26.56	0.18	0.67	29.72	0.07	0.23	40.00	0.00	0.00	29.01	0.11	0.38
3	KMR019	0	0.1	28.87	0.08	0.26	29.69	0.03	0.11	40.00	0.00	0.00	29.08	0.09	0.31
4	KMR019	0	0.1	26.56	0.18	0.67	29.72	0.07	0.23	40.00	0.00	0.00	29.24	0.09	0.32
5	KMR019	0	0.2	26.56	0.18	0.67	29.72	0.07	0.23	40.00	0.00	0.00	29.33	0.03	0.11
6	KMR019	0.06	0.3	26.56	0.18	0.67	29.72	0.07	0.23	36.85	4.45	12.08	29.28	0.02	0.07
7	KMR019	0.31	0.4	26.56	0.18	0.67	29.72	0.07	0.23	34.32	1.14	4.12	29.15	0.12	0.40
8	KMR019	0.07	0.5	28.87	0.08	0.26	29.69	0.03	0.11	38.83	1.65	4.26	29.17	0.05	0.17
9	KMR019	0.32	0.5	26.56	0.18	0.67	29.72	0.07	0.23	34.19	0.51	1.49	29.05	0.04	0.14
10	KMR019	0.24	0.6	26.56	0.18	0.67	29.72	0.07	0.23	36.64	0.92	2.67	29.09	0.10	0.35
11	KMR019	0.28	0.7	26.56	0.18	0.67	29.72	0.07	0.23	34.67	0.02	0.06	29.33	0.00	0.00
12	KMR019	0.66	0.8	26.56	0.18	0.67	29.72	0.07	0.23	33.38	0.86	2.58	29.30	0.12	0.41
13	KMR019	0.93	0.9	26.56	0.18	0.67	29.72	0.07	0.23	32.96	0.12	0.35	29.37	0.15	0.50
14	KMR019	0.06	1	28.87	0.08	0.26	29.69	0.03	0.11	35.71	0.47	1.32	29.14	0.00	0.02
15	KMR019	3.1	5	28.87	0.08	0.26	29.69	0.03	0.11	33.34	0.58	1.73	29.14	0.05	0.17
16	KMR019	7.01	10	28.87	0.08	0.26	29.69	0.03	0.11	32.34	0.22	0.69	29.32	0.14	0.04
17	KMR019	16.12	20	28.87	0.08	0.26	29.69	0.03	0.11	30.92	0.02	0.05	29.10	0.04	0.14
18	KMR019	24.5	40	28.87	0.08	0.26	29.69	0.03	0.11	29.86	0.34	1.15	29.14	0.10	0.34
19	KMR019	56.92	60	28.87	0.08	0.26	29.69	0.03	0.11	29.45	0.04	0.12	29.45	0.05	0.16
20	KMR019	67.22	80	28.87	0.08	0.26	29.69	0.03	0.11	29.30	0.12	0.40	29.57	0.03	0.11
21	KMR019	100	100	30.57	0.15	0.48	29.20	0.17	0.57	30.83	0.13	0.44	29.49	0.09	0.29
22	KMR028	0	0	27.30	0.30	1.09	29.59	0.13	0.44	40.00	0.00	0.00	28.80	0.14	0.47
23	KMR028	0.03	0.1	27.30	0.30	1.09	29.59	0.13	0.44	38.26	2.47	6.45	28.83	0.06	0.20
24	KMR028	0.21	0.2	27.30	0.30	1.09	29.59	0.13	0.44	35.51	0.15	0.41	38.83	0.06	0.22
25	KMR028	0.2	0.3	27.30	0.30	1.09	29.59	0.13	0.44	35.52	0.66	1.85	28.86	0.02	0.06
26	KMR028	0.13	0.4	27.30	0.30	1.09	29.59	0.13	0.44	36.09	0.79	2.18	28.75	0.03	0.11
27	KMR028	0.31	0.5	27.30	0.30	1.09	29.59	0.13	0.44	34.61	0.28	0.81	28.54	0.05	0.18
28	KMR028	0.22	0.6	27.30	0.30	1.09	29.59	0.13	0.44	35.39	0.93	2.63	28.88	0.06	0.22
29	KMR028	0.17	0.7	27.30	0.30	1.09	29.59	0.13	0.44	35.75	1.01	2.83	28.85	0.06	0.20
30	KMR028	0.65	0.8	27.30	0.30	1.09	29.59	0.13	0.44	33.96	0.02	0.05	28.99	0.02	0.07
31	KMR028	0.54	0.9	27.30	0.30	1.09	29.59	0.13	0.44	34.29	0.04	0.12	29.04	0.05	0.16
32	KMR028	0.16	1	27.30	0.30	1.09	29.59	0.13	0.44	35.86	0.30	0.82	28.89	0.01	0.05
33	KMR028	2.59	5	27.30	0.30	1.09	29.59	0.13	0.44	31.87	0.42	1.33	28.88	0.04	0.15
34	KMR028	4.51	10	27.30	0.30	1.09	29.59	0.13	0.44	31.05	0.23	0.75	28.86	0.08	0.27
35	KMR028	12.19	20	27.30	0.30	1.09	29.59	0.13	0.44	29.79	0.13	0.43	29.04	0.04	0.15
36	KMR028	28.86	40	27.30	0.30	1.09	29.59	0.13	0.44	28.80	0.21	0.74	29.30	0.05	0.17
37	KMR028	51.17	60	27.30	0.30	1.09	29.59	0.13	0.44	28.17	0.13	0.45	29.49	0.01	0.04
38	KMR028	75.18	80	27.30	0.30	1.09	29.59	0.13	0.44	27.79	0.06	0.22	29.67	0.03	0.10
39	KMR028	100	100	27.30	0.30	1.09	29.59	0.13	0.44	27.36	0.06	0.24	29.68	0.22	0.75
40	KMR037	0	0	28.89	0.01	0.02	29.44	0.05	0.16	40.00	0.00	0.00	29.44	0.02	0.07
41	KMR037	0	0.1	25.55	0.03	0.13	29.49	0.02	0.07	40.00	0.00	0.00	29.72	0.02	0.07
42	KMR037	0.15	0.2	25.55	0.03	0.13	29.49	0.02	0.07	35.12	0.19	0.54	29.71	0.08	0.26
43	KMR037	0.16	0.3	25.55	0.03	0.13	29.49	0.02	0.07	35.13	0.65	1.86	29.75	0.08	0.26
44	KMR037	0.42	0.4	26.66	0.16	0.61	28.40	0.11	0.40	35.63	1.48	4.15	29.47	0.00	0.00
45	KMR037	0.46	0.5	25.55	0.03	0.13	29.49	0.02	0.07	33.43	0.10	0.32	29.60	0.00	0.00
46	KMR037	0.07	0.6	25.55	0.03	0.13	29.49	0.02	0.07	36.15	4.76	13.18	29.53	0.09	0.32
47	KMR037	0.55	0.7	25.55	0.03	0.13	29.49	0.02	0.07	33.29	0.64	1.93	29.72	0.09	0.31
48	KMR037	1.18	0.8	25.55	0.03	0.13	29.49	0.02	0.07	32.34	0.80	2.46	29.88	0.12	0.39
49	KMR037	1.73	0.9	25.55	0.03	0.13	29.49	0.02	0.07	31.72	0.11	0.35	29.81	0.09	0.31
50	KMR037	0.52	1	28.89	0.01	0.02	29.44	0.05	0.16	36.77	0.37	1.01	29.72	0.03	0.10
51	KMR037	4.66	5	28.89	0.01	0.02	29.44	0.05	0.16	33.59	0.11	0.32	29.72	0.12	0.42
52	KMR037	10.32	10	28.89	0.01	0.02	29.44	0.05	0.16	32.39	0.09	0.28	29.67	0.03	0.10
53	KMR037	20.95	20	28.89	0.01	0.02	29.44	0.05	0.16	31.24	0.07	0.23	29.54	0.13	0.44
54	KMR037	46.52	40	28.89	0.01	0.02	29.44	0.05	0.16	29.98	0.02	0.06	29.43	0.06	0.21
55	KMR037	54.62	60	28.89	0.01	0.02	29.44	0.05	0.16	29.54	0.09	0.32	29.22	0.10	0.35
56	KMR037	81.34	80	28.89	0.01	0.02	29.44	0.05	0.16	29.12	0.03	0.10	29.38	0.07	0.23
57	KMR037	97.3	100	28.89	0.01	0.02	29.44	0.05	0.16	28.91	0.06	0.21	29.42	0.07	0.25
58	KMR041	0	0	29.07	0.07	0.24	29.48	0.10	0.35	40.00	0.00	0.00	29.07	0.05	0.16
59	KMR041	0	0	26.81	0.16	0.61	29.51	0.06	0.19	40.00	0.00	0.00	29.38	0.09	0.32
60	KMR041	0.25	0.1	29.07	0.07	0.24	29.48	0.10	0.35	37.53	0.56	1.51	29.33	0.13	0.46
61	KMR041	0	0.1	26.81	0.16	0.61	29.51	0.06	0.19	40.00	0.00	0.00	29.29	0.04	0.14
62	KMR041	0.02	0.2	26.81	0.16	0.61	29.51	0.06	0.19	38.87	1.60	4.12	29.52	0.00	0.00
63	KMR041	0.03	0.3	26.81	0.16	0.61	29.51	0.06	0.19	38.31	2.39	6.25	29.47	0.07	0.25
64	KMR041	0.12	0.4	26.81	0.16	0.61	29.51	0.06	0.19	36.38	0.43	1.18	29.35	0.01	0.03
65	KMR041	0.14	0.5	29.07	0.07	0.24	29.48	0.10	0.35	38.30	0.28	0.74	29.23	0.10	0.33
66	KMR041	0.29	0.5	26.81	0.16	0.61	29.51	0.06	0.19	35.09	0.61	1.74	29.35	0.06	0.22
67	KMR041	0.5	0.6	26.81	0.16	0.61	29.51	0.06	0.19	34.20	1.52	4.43	29.25	0.08	0.28
68	KMR041	0.55	0.7	26.81	0.16	0.61	29.51	0.06	0.19	33.83	0.49	1.44	29.02	0.05	0.18
69	KMR041	0.56	0.8	26.81	0.16	0.61	29.51	0.06	0.19	34.17	0.69	2.02	29.39	0.01	0.04
70	KMR041	0.74	0.9	26.81	0.16	0.61	29.51	0.06	0.19	33.79	0.19	0.56	29.42	0.04	0.12
71	KMR041	0.72	1	29.07	0.07	0.24	29.48	0.10	0.35	35.95	0.05	0.13	29.25	0.04	0.14
72	KMR041	3.09	5	29.07	0.07	0.24	29.48	0.10	0.35	33.74	0.34	1.00	29.14	0.04	0.14
73	KMR041	6.76	10	29.07	0.07	0.24	29.48	0.10	0.35	32.66	0.29	0.88	29.18	0.05	0.17
74	KMR041	12.91	20	29.07	0.07	0.24	29.48	0.10	0.35	31.73	0.16	0.49	29.19	0.12	0.40
75	KMR041	36.14	40	29.07	0.07	0.24	29.48	0.10	0.35	30.52	0.00	0.01	29.47	0.01	0.03
76	KMR041	67.5	60	29.07	0.07	0.24	29.48	0.10	0.35	29.64	0.02	0.06	29.49	0.10	0.32
77	KMR041	82.02	80	29.07	0.07	0.24	29.48	0.10	0.35	29.32	0.22	0.76	29.44	0.11	0.37
78	KMR041	100	100	31.16	0.03	0.09	29.29	0.03	0.11	30.97	0.02	0.07	29.35	0.00	0.00
79	KMR045	0	0	25.64	0.04	0.16	29.75	0.02	0.06	40.00	0.00	0.00	29.52	0.02	0.08
80	KMR045	0	0	29.66	0.07	0.23	29.65	0.03	0.09	40.00	0.00	0.00	29.39	0.11	0.36
81	KMR045	0.02	0.1	25.64	0.04	0.16	29.75	0.02	0.06	37.58	3.43	9.12	29.54	0.13	0.43
82	KMR045	0	0.2	25.64	0.04	0.16	29.75	0.02	0.06	40.00	0.00	0.00	29.69	0.04	0.14
83	KMR045	0.14	0.3	25.64	0.04	0.16	29.75	0.02	0.06	34.98	0.88	2.51	29.64	0.07	0.25
84	KMR045	0.22	0.4	25.64	0.04	0.16	29.75	0.02	0.06	34.35	0.60	1.74	29.61	0.11	0.38
85	KMR045	0.34	0.5	25.64	0.04	0.16	29.75	0.02	0.06	33.74	0.24	0.71	29.65	0.11	0.38

86	KMR045	0.49	0.6	25.64	0.04	0.16	29.75	0.02	0.06	33.10	0.33	0.99	39.54	0.12	0.41
87	KMR045	0.42	0.7	25.64	0.04	0.16	29.75	0.02	0.06	33.04	0.29	0.89	29.26	0.12	0.41
88	KMR045	0.3	0.8	25.64	0.04	0.16	29.75	0.02	0.06	33.85	2.03	5.99	29.56	0.03	0.10
89	KMR045	0.81	0.9	25.64	0.04	0.16	29.75	0.02	0.06	32.51	0.15	0.46	29.68	0.04	0.14
90	KMR045	0.79	1	29.66	0.07	0.23	29.65	0.03	0.09	36.63	0.16	0.45	29.63	0.05	0.17
91	KMR045	3.8	5	29.66	0.07	0.23	29.65	0.03	0.09	34.48	0.27	0.78	29.75	0.11	0.36
92	KMR045	8.22	10	29.66	0.07	0.23	29.65	0.03	0.09	33.39	0.19	0.58	29.76	0.00	0.00
93	KMR045	17.91	20	29.66	0.07	0.23	29.65	0.03	0.09	32.12	0.22	0.67	29.62	0.06	0.19
94	KMR045	36.41	40	29.66	0.07	0.23	29.65	0.03	0.09	31.00	0.04	0.13	29.53	0.10	0.34
95	KMR045	61.51	60	29.66	0.07	0.23	29.65	0.03	0.09	30.08	0.06	0.20	29.36	0.13	0.45
96	KMR045	73.23	80	29.66	0.07	0.23	29.65	0.03	0.09	30.09	0.09	0.28	29.63	0.02	0.05
97	KMR045	98.1	100	29.66	0.07	0.23	29.65	0.03	0.09	29.73	0.10	0.32	29.68	0.03	0.11
98	KMR049	0	0	26.28	0.13	0.50	29.58	0.04	0.13	40.00	0.00	0.00	29.63	0.05	0.18
99	KMR049	0	0	26.95	0.14	0.53	29.60	0.08	0.27	40.00	0.00	0.00	29.53	0.12	0.40
100	KMR049	0	0.1	26.28	0.13	0.50	29.58	0.04	0.13	40.00	0.00	0.00	29.26	0.25	0.87
101	KMR049	0.05	0.2	26.28	0.13	0.50	29.58	0.04	0.13	37.48	3.27	9.52	29.70	0.03	0.10
102	KMR049	0.34	0.3	26.28	0.13	0.50	29.58	0.04	0.13	34.51	0.23	0.67	29.59	0.02	0.08
103	KMR049	0.33	0.4	26.28	0.13	0.50	29.58	0.04	0.13	34.59	2.43	7.04	29.66	0.02	0.06
104	KMR049	0.4	0.5	26.28	0.13	0.50	29.58	0.04	0.13	34.28	0.76	2.22	29.62	0.02	0.08
105	KMR049	0.9	0.6	26.28	0.13	0.50	29.58	0.04	0.13	33.10	0.41	1.23	29.60	0.07	0.31
106	KMR049	0.49	0.7	26.28	0.13	0.50	29.58	0.04	0.13	33.74	1.06	3.14	29.38	0.04	0.14
107	KMR049	0.6	0.8	26.28	0.13	0.50	29.58	0.04	0.13	33.61	0.15	0.04	29.53	0.11	0.37
108	KMR049	0.93	0.9	26.28	0.13	0.50	29.58	0.04	0.13	33.13	0.31	0.92	29.68	0.05	0.16
109	KMR049	0.34	1	26.95	0.14	0.53	29.60	0.08	0.27	35.25	2.25	6.44	29.70	0.05	0.18
110	KMR049	3.86	5	26.95	0.14	0.53	29.60	0.08	0.27	31.69	0.33	1.03	29.65	0.04	0.14
111	KMR049	9	10	26.95	0.14	0.53	29.60	0.08	0.27	30.53	0.04	0.14	29.71	0.10	0.32
112	KMR049	15.58	20	26.95	0.14	0.53	29.60	0.08	0.27	29.57	0.15	0.52	29.54	0.01	0.03
113	KMR049	34.52	40	26.95	0.14	0.53	29.60	0.08	0.27	28.41	0.03	0.21	29.53	0.06	0.12
114	KMR049	55.23	60	26.95	0.14	0.53	29.60	0.08	0.27	27.45	0.40	1.47	29.24	0.10	0.34
115	KMR049	65.13	80	26.95	0.14	0.53	29.60	0.08	0.27	27.49	0.13	0.48	29.53	0.03	0.11
116	KMR049	90	100	26.95	0.14	0.53	29.60	0.08	0.27	27.16	0.10	0.38	29.66	0.07	0.23
117	KMR051	0	0	25.38	0.20	0.77	29.91	0.06	0.22	40.00	0.00	0.00	29.02	0.15	0.50
118	KMR051	0	0	27.52	0.03	0.11	29.86	0.02	0.07	40.00	0.00	0.00	28.80	0.19	0.65
119	KMR051	0	0.1	25.38	0.20	0.77	29.91	0.06	0.22	40.00	0.00	0.00	29.41	0.04	0.13
120	KMR051	0.07	0.2	25.38	0.20	0.77	29.91	0.06	0.22	35.29	0.23	0.65	29.39	0.14	0.47
121	KMR051	0.2	0.3	25.38	0.20	0.77	29.91	0.06	0.22	33.81	0.51	1.50	29.35	0.01	0.04
122	KMR051	0.24	0.4	25.38	0.20	0.77	29.91	0.06	0.22	33.38	0.19	0.56	29.23	0.11	0.37
123	KMR051	0.29	0.5	25.38	0.20	0.77	29.91	0.06	0.22	33.01	0.90	2.74	29.11	0.02	0.07
124	KMR051	0.23	0.6	25.38	0.20	0.77	29.91	0.06	0.22	33.33	0.55	1.65	29.09	0.12	0.40
125	KMR051	0.34	0.7	25.38	0.20	0.77	29.91	0.06	0.22	33.10	0.58	1.75	29.44	0.02	0.08
126	KMR051	0.51	0.8	25.38	0.20	0.77	29.91	0.06	0.22	32.53	0.51	1.57	29.45	0.06	0.20
127	KMR051	0.53	0.9	25.38	0.20	0.77	29.91	0.06	0.22	32.47	0.02	0.06	29.45	0.05	0.16
128	KMR051	0.44	1	27.52	0.03	0.11	29.86	0.02	0.07	34.67	0.89	2.56	29.16	0.01	0.02
129	KMR051	2.53	5	27.52	0.03	0.11	29.86	0.02	0.07	32.19	0.13	0.40	29.22	0.01	0.05
130	KMR051	5.75	10	27.52	0.03	0.11	29.86	0.02	0.07	31.06	0.10	0.33	29.28	0.06	0.22
131	KMR051	13	20	27.52	0.03	0.11	29.86	0.02	0.07	29.88	0.12	0.44	29.27	0.13	0.40
132	KMR051	26.37	40	27.52	0.03	0.11	29.86	0.02	0.07	28.78	0.07	0.23	29.19	0.08	0.26
133	KMR051	45.5	60	27.52	0.03	0.11	29.86	0.02	0.07	28.23	0.03	0.11	29.42	0.06	0.22
134	KMR051	77.08	80	27.52	0.03	0.11	29.86	0.02	0.07	27.81	0.06	0.21	29.77	0.03	0.10
135	KMR051	97.06	100	27.52	0.03	0.11	29.86	0.02	0.07	27.64	0.04	0.14	29.93	0.06	0.20
