# Supplementary material for: Evaluation of a quantitative PCR-based method for chimerism analysis of Japanese donor/recipient pairs
Source: Sci Rep. 2022 Dec 9;12:21328. doi: 10.1038/s41598-022-25878-9 (PMC9734659; doi:10.1038/s41598-022-25878-9)
Supplement: Supplementary file 6 — Supplementary Information 6. [file 41598_2022_25878_MOESM6_ESM.pdf]

Supplemental Table 9. Raw mean Cq values and recipient chimerism evaluated by KMRtrack kit with KMRengine and previous methods

UID	KMR						Previous method	
	Marker	Reference Sample Mean Cq		Monitoring Sample Mean Cq		Recipient%	Method	Recipient%
		Informative marker	Reference assay	Informative marker	Reference assay			
4352	KMR041	24.85	26.04	33.77	25.93	0.19	STR	0
4352	KMR052	26.04	26.04	33.67	25.93	0.46	STR	0
4454	KMR045	25.48	26.18	35.01	26.91	0.22	STR	0
4532	KMR019	25.28	25.76	34.53	26.08	0.21	STR	0
4544	KMR041	27.83	26.65	27.10	25.75	89.03	STR	94
4544	KMR013	30.53	28.64	31.27	28.99	76.10	STR	94
4545	KMR051	25.50	26.65	32.05	29.49	7.71	STR	12
4558	KMR037	31.37	31.57	35.28	31.28	5.46	STR	18
4558	KMR052	28.98	28.03	32.47	27.77	7.44	STR	18
4564	KMR037	29.88	30.11	30.70	29.79	45.21	STR	34
4564	KMR013	26.51	25.74	28.11	25.73	32.65	STR	34
4643	KMR041	28.93	26.94	40.00	32.77	0.00	STR	0
4652	KMR037	27.59	26.70	34.73	25.87	0.40	STR	0
4652	KMR019	27.80	30.13	35.43	30.14	0.51	STR	0
4686	KMR041	29.19	28.03	40.00	27.80	0.00	STR	0
4686	KMR048	30.96	28.03	40.00	27.80	0.00	STR	0
4694	KMR019	25.91	25.86	30.93	26.14	3.74	STR	30
4719	KMR028	27.01	26.21	29.37	25.79	14.60	STR	15
4876	KMR028	27.37	27.83	33.62	25.88	0.36	STR	0
4921	KMR050	27.16	30.09	35.22	29.50	0.28	STR	0
4921	KMR045	24.43	29.22	33.26	29.03	0.19	STR	0
4921	KMR051	26.07	29.22	33.92	29.03	0.54	STR	0
4934	KMR019	24.37	25.00	30.03	26.19	4.56	STR	13
4946	KMR050	27.16	30.09	34.32	29.10	0.35	STR	0
4946	KMR045	24.43	29.22	33.34	28.53	0.13	STR	0
4946	KMR051	26.07	29.22	34.65	28.53	0.23	STR	0
4976	KMR050	27.16	30.09	34.56	29.42	0.37	STR	0
4976	KMR045	24.43	29.22	37.74	28.85	0.06	STR	0
4976	KMR051	26.07	29.22	34.16	28.53	0.40	STR	0
4987	KMR041	27.13	25.57	34.42	36.49	1.20	STR	6
4987	KMR051	26.82	28.38	33.25	28.90	1.67	STR	6
5009	KMR051	25.68	25.48	33.02	28.26	0.81	STR	13
5015	KMR050	27.16	30.09	35.12	29.26	0.23	STR	0
5015	KMR045	24.43	29.22	33.13	29.10	0.22	STR	0
5015	KMR051	26.07	29.22	33.81	29.10	0.59	STR	0
5065	KMR037	26.63	26.21	27.10	26.02	63.35	STR	70
5065	KMR038	28.16	27.53	30.53	29.55	78.66	STR	70
5085	KMR045	32.43	32.06	29.11	28.77	100.00	STR	100
5108	KMR050	27.16	30.09	27.74	29.78	53.69	STR	38
5108	KMR045	24.43	29.22	26.51	29.20	23.15	STR	38
5108	KMR051	26.07	29.22	27.92	29.20	38.48	STR	38
5119	KMR041	28.63	26.89	29.26	27.97	100.00	STR	93
5125	KMR045	27.49	28.28	33.02	27.99	1.77	STR	2
5125	KMR041	30.29	31.06	35.09	30.22	2.01	STR	2
5135	KMR037	27.15	26.19	31.52	25.21	2.45	STR	5
5166	KMR045	24.71	26.76	28.41	27.84	16.32	STR	15
5168	KMR050	27.16	30.09	27.09	29.29	60.35	STR	54
5168	KMR045	24.43	29.22	25.44	28.92	40.13	STR	54
5168	KMR051	26.07	29.22	27.06	28.92	57.70	STR	54
5172	KM0R37	30.93	31.00	33.86	31.07	13.79	STR	12
5289	KMR049	25.91	28.44	31.15	27.11	1.05	STR	0
5387	KMR049	26.74	27.16	35.84	27.86	0.30	STR	0
5387	KMR040	32.14	30.81	40.00	30.73	0.00	STR	0
5403	KMR028	27.13	27.33	35.26	26.75	0.24	STR	0
5403	KMR009	31.66	29.18	40.00	28.66	0.00	STR	0
5556	KMR045	29.93	29.33	39.41	29.88	0.21	STR	0
5556	KMR049	27.74	28.62	36.54	29.14	0.32	STR	0
5578	KMR051	26.56	26.15	34.86	26.09	0.31	SNP-qPCR (R)	0
5728	KMR051	26.76	26.07	32.50	26.18	2.02	SNP-qPCR (R)	3
5764	KMR019	26.55	27.18	26.29	25.33	33.18	SNP-qPCR (R)	50
5803	KMR045	26.42	25.93	27.99	27.58	100.00	SNP-qPCR (R)	97
5803	KMR037	29.38	29.49	30.24	30.24	97.94	STR	97
5881	KMR037	25.74	26.32	26.85	26.09	39.22	SNP-qPCR (R)	57
